# Surface-mediated assembly, polymerization and degradation of thiophene-based monomers†
†Electronic supplementary information (ESI) available: Additional STM, XPS and DFT results, higher size version of Fig. 2, 5, 8, and a discussion of chirality. See DOI: 10.1039/c8sc05267k.


**DOI:** 10.1039/c8sc05267k

**Published:** 2019-04-16

**Authors:** G. Galeotti, F. De Marchi, T. Taerum, L. V. Besteiro, M. El Garah, J. Lipton-Duffin, M. Ebrahimi, D. F. Perepichka, F. Rosei

**Affiliations:** a Centre Energie, Matériaux et Télécommunications , Institut National de la Recherche Scientifique , 1650 Boulevard Lionel-Boulet , Varennes , Québec , Canada J3X 1S2 . Email: maryam.ebrahimi@emt.inrs.ca ; Email: rosei@emt.inrs.ca; b Istituto di Struttura della Materia , CNR , Via Fosso del Cavaliere 100 , 00133 Roma , Italy; c Department of Chemistry , McGill University , 801 Sherbrooke Street West , Montreal , Quebec , Canada H3A 0B8 . Email: dmitrii.perepichka@mcgill.ca; d Institute of Fundamental and Frontier Science , University of Electronic Science and Technology of China , Chengdu 610054 , PR China; e Institute for Future Environments , Queensland University of Technology (QUT) , 2 George Street , Brisbane , 4001 QLD , Australia

## Abstract

Ullmann coupling of halogenated aromatics is widely used in on-surface synthesis of two-dimensional (2D) polymers and graphene nanoribbons. It stands out among other reactions for regioselectively connecting aromatic monomers into 1D and 2D π-conjugated polymers, whose final structure and properties are determined by the initial building blocks. Thanks to their exceptional electronic properties, thiophene-containing monomers are frequently used for the synthesis of various conjugated materials. On the other hand, their use in on-surface polymerization is hampered by the possibility of ring opening when adsorbed on metal surfaces. In the present work, we mapped the temperature regime for these two competing reactions by investigating the adsorption of a thiophene-based prochiral molecule using scanning tunneling microscopy, X-ray photoelectron spectroscopy and density functional theory calculations. We followed the formation of organometallic (OM) networks, their evolution into covalent structures and the competition between C–C coupling and thiophene ring opening. The effect of surface reactivity was explored by comparing the adsorption on three (111) coinage metal substrates, namely Au, Ag and Cu. While outlining strategies to minimize the ring opening reaction, we found that the surface temperature during deposition is of paramount importance for the preparation of 2D OM networks, greatly enhancing the overall ordering of the product by depositing on hot Ag surface. Notably, the same protocol permits the creation of OM structures on the air-stable Au surface, thereby allowing the synthesis and application of 2D OM networks outside the ultra-high vacuum environment.

## Abstract 2

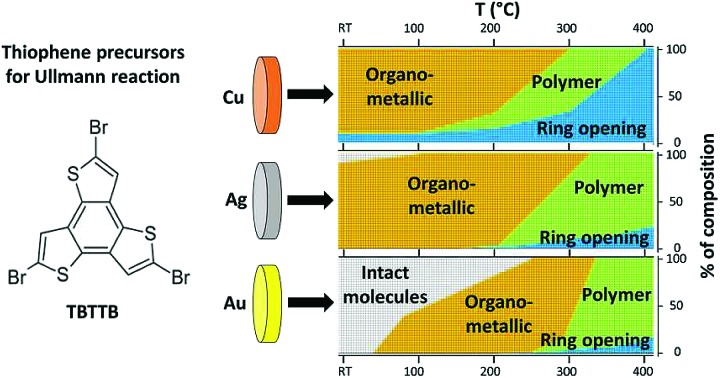
Temperature mapping of the different molecular phases of tribromoterthienobenzene on (111) coinage metals.

## Introduction

Two-dimensional (2D) materials have been extensively studied due to their unprecedented properties arising from reduced dimensionality.^1^ Thanks to their tunable band gap, π-conjugated 2D polymers^2^,^3^ constitute an emerging class of 2D materials with potential applications in nanoelectronics and optoelectronics.^4^–^6^ However, their synthesis is challenging due to the difficulty in controlling the dimensionality during conventional solution-based polymerization, while chemical vapor deposition methods^7^ require an extremely high chemoselectivity considering the molecular complexity of the monomers.^8^,^9^

One promising approach to synthesize 2D polymers is based on the self-assembly of reactive molecules on atomically flat surfaces,^10^ followed by polymerization.^11^ Several reactions have been explored for this purpose, including Sonogashira coupling of haloaromatic precursors with terminal alkynes,^12^ oxidative terminal alkyne (Glaser–Hay) coupling,^13^ photo/electro-initiated polymerization of diacetylene derivatives,^11^ decarboxylative coupling^14^ and electro-oxidative polymerization of thiophenes.^15^ Among these, on-surface Ullmann coupling is the most broadly applicable and frequently used reaction for the synthesis of 2D π-conjugated polymers.^16^–^20^ The reaction is catalyzed by coinage metal (Cu, Ag, and Au) surfaces and involves the dissociation of a carbon–halogen bond resulting in an organometallic (OM) phase^21^,^22^ which is followed by carbon–carbon coupling.^23^ The main reasons for the widespread use of Ullmann coupling are its efficiency and wide scope. By changing the structure of the monomer and the position of the halogen along the molecular backbone, it is possible to control the polymer's dimensionality and topology.^19^

The required polymerization temperature is dictated by the chosen substrate and halogen, and a multistep hierarchical growth process can be engineered by including different types of halogens.^24^,^25^ The substrate affects both the thermodynamics of the system and the reaction kinetics, driving the reaction along different pathways, and changing the relative stability of its intermediates (*e.g.*, destabilizing the OM structures).^26^,^27^

A variety of building blocks have been used in the on-surface synthesis of π-conjugated 2D polymers. The structure of the monomers and their network connectivity control electron delocalization, band gap and charge carrier mobility.^28^–^30^ Incorporating heteroatoms into the monomer's backbone is a well-established approach to modify the polymer's properties, and a useful tool in tuning the doped carbon materials towards applications in catalysis, energy and semiconducting devices.^31^ In this context, thiophene-containing monomers are particularly promising for the synthesis of π-conjugated 2D polymers, due to their synthetic feasibility, structural diversity, highly efficient π-conjugation, and already well established applications of solution-processable (1D) polythiophenes in semiconducting devices.^32^,^33^

Nevertheless, on-surface polymerization of thiophene monomers *via* Ullmann coupling is challenging. In fact, the sulphur–metal interaction is found to be too strong on nickel and copper surfaces, resulting in partial or complete scission of C–S bonds.^18^,^34^–^36^ On the other hand, it was recently reported that 2,5-dichloro(3,4-ethylenedioxythiophene) could be polymerized into linear PEDOT without ring openings on the Ag surface (but not on Cu or Au).^37^ Thus, the extent of desulfurization and its dependence on the substrate, the molecular structure and the reaction temperature need to be understood in order to access well-defined sulfur-doped carbon nanomaterials.

**Fig. 1:**
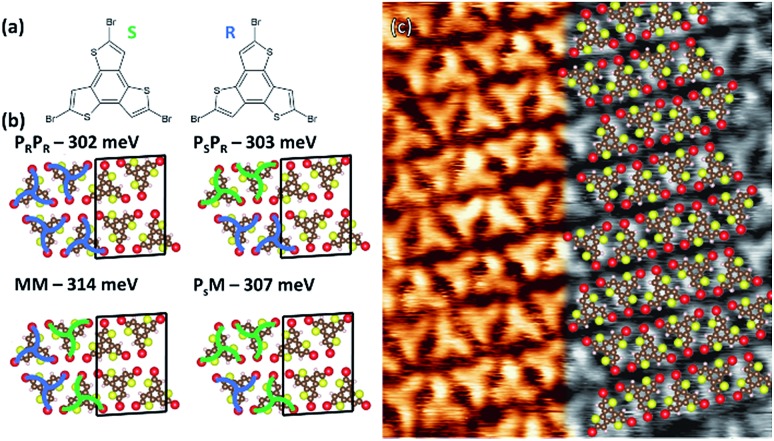
(a) Molecular structures of *S* and *R* TBTTB enantiomers; (b) possible enantiomeric configurations for TBTTB adsorbed on Au(111) at RT and their calculated cohesive energy per molecule. Black boxes represent the unit cells. (c) 7.5 × 7.5 nm^2^ STM image of TBTTB on Au(111) superimposed with the MM structure calculated by DFT (*I*_t_ = 0.63 nA; *V*_t_ = 1.25 V).

Here we present a comparative study of self-assembly and two-step reactivity of a tridentate tribromoterthienobenzene (TBTTB, Fig. 1a) on Au(111), Ag(111) and Cu(111), using scanning tunneling microscopy (STM) and X-ray photoelectron spectroscopy (XPS), supported by density functional theory (DFT) calculations. The terthienobenzene (TTB) core^38^ of this monomer presents a higher S/C ratio in comparison to other previously studied monomers,^18^,^39^,^40^ and has been used as a building block in several semiconducting materials,^41^ including star-shaped oligomers,^42^ linear conjugated polymers,^43^ and 3D microporous polymers.^44^–^46^

By depositing the molecules over a substrate kept at room temperature (RT) we obtain 2D self-assembled molecular networks on Au, and OM structures on Ag and Cu. Our results demonstrate that while annealing at elevated temperatures is necessary to trigger Ullmann coupling, it also causes the adsorbates to undergo competing ring opening reactions, due to the high affinity of sulphur with coinage metals and relatively low C–S bond dissociation energy (3.24 eV for C–S *vs.* 3.05 eV for C–Br).^47^ We map out the temperature dependence of these two competing reactions on the three investigated surfaces, and show that the activation barrier for the ring opening exceeds the barrier for dehalogenation for all three surfaces.^26^,^48^

By depositing directly on a heated substrate, we show that it is possible to improve the overall ordering of the 2D OM network on Ag and to obtain similar hexagonal networks on Au. While common for Ag and Cu surfaces,^27^,^49^ Ullmann coupling on Au rarely yields stable OM intermediates. Although short 1D Au-bridged OM chains have been observed in recent studies,^50^–^53^ the formation of 2D OM networks on gold has not been previously reported.

## Results and discussion

### TBTTB on Au(111)

**Fig. 2:**
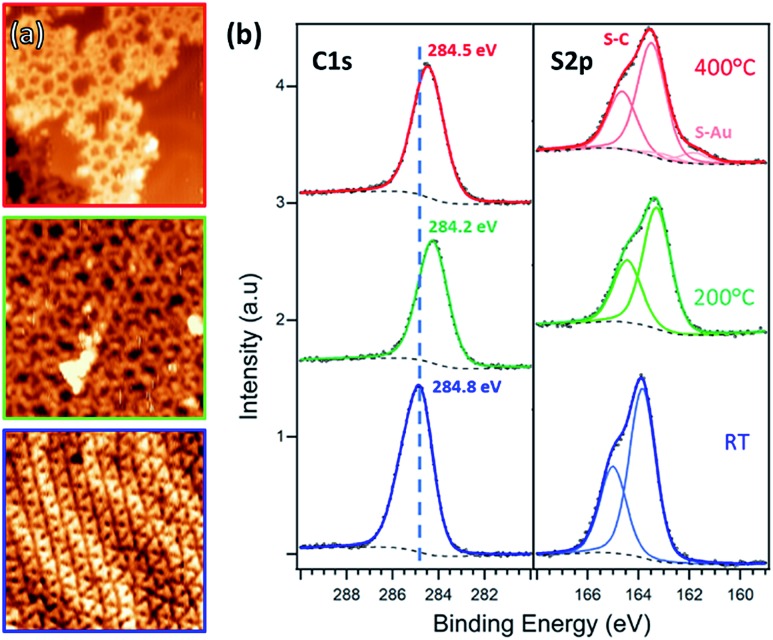
(a) STM images and (b) XPS C 1s and S 2p spectra of TBTTB deposited on Au(111) at RT (blue, *I*_t_ = 0.13 nA; *V*_t_ = –0.56 V), and sequentially annealed to 200 °C (green, *I*_t_ = 0.86 nA; *V*_t_ = –2.01 V) and 400 °C (red, *I*_t_ = 0.25 nA; *V*_t_ = 1.25 V).

**Fig. 3:**
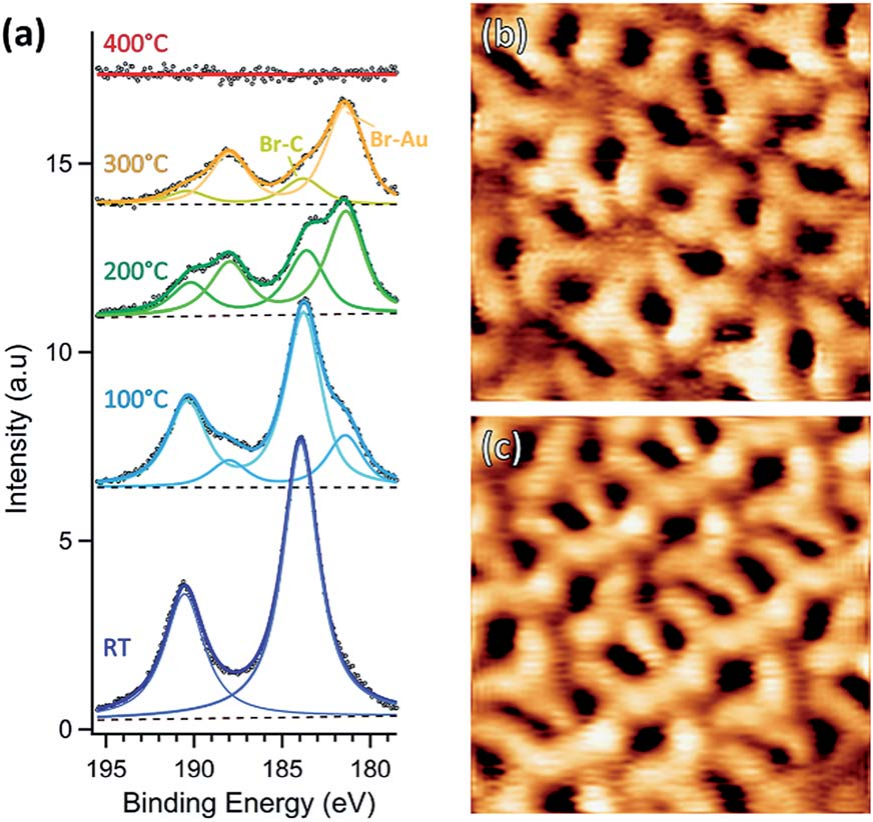
(a) Br 3p XPS spectra of 1 ML of TBTTB at various temperatures. (b and c) 4.5 × 4.5 nm^2^ STM images of TBTTB on Au(111) annealed @ 100 °C. The SAMNs formed at RT are partially warped upon annealing, and patches of hexagonal (b) or linear (c) domains are found on the surface ((b) *I*_t_ = –0.29 nA; *V*_t_ = –0.45 V; (c) *I*_t_ = –0.12 nA; *V*_t_ = –0.31 V).

After deposition on Au at RT, the molecules organize into a close-packed 2D row structure (Fig. 1c). The unit cell, with dimensions of (1.73 ± 0.05) × (2.45 ± 0.05) nm^2^ and an angle of 87 ± 3° between two vectors, contains four molecules in two rows of alternating *R*/*S* enantiomers (the identification of the enantiomers is described in the ESI, Section 1†). To confirm this assignment, we performed DFT gas phase calculations (PBE-GGA with D3 dispersion correction), calculating all the possible combinations of enantiopure (*P*_s_ or *P*_R_) and racemic (*M*) rows (Fig. 1b and S1†). The total cohesive energies indicate that the racemic networks are slightly more stable (Δ*E* ≈ 0.05 eV per unit cell) than the homo-enantiomeric assemblies. As such, the simulations point toward the MM combination as the most stable assembly, as inferred from STM images. The herringbone reconstruction of the Au(111) surface is maintained (Fig. 2a, blue frame), consistent with weak molecule–substrate interactions.^50^,^54^ XPS analysis shows a Br 3p doublet at binding energies (BEs) of 183.7 eV (Br 3p_3/2_) and 190.2 eV (Br 3p_1/2_) and a S 2p doublet at BEs of 163.9 eV (S 2p_3/2_) and 165.1 eV (S 2p_1/2_), as expected for Br–C and S–C bonds, confirming that the molecules are intact (Fig. 2b and 3a).^18^

Annealing the chiral phase on Au(111) reduces the level of order of the molecular network. Starting from 100 °C, additional arrangements are observed to coexist with the intact RT phase (Fig. 3b, c and S2†). XPS shows the emergence of a new Br 3p_3/2_ peak at BE of 181.4 eV (Br–Au), due to partial Br–C dissociation (Fig. 3a). We infer that the new arrangements are produced by the presence of partially debrominated monomers and the dissociated Br atoms that locally perturb the self-assembled structures (Fig. 3b and c). As the temperature increases, the Br–Au peak grows at the expense of the Br–C signal, while Br atoms progressively desorb leaving the surface bromine-free at 400 °C (Fig. 3c).

After annealing for 30 minutes at 200 °C, this gradual dehalogenation results in the formation of disordered OM networks (Fig. 2a, green frame and S3†), with the C and S peaks shifting toward lower BEs (S 2p_3/2_ at 163.5 eV, C 1s at 284.2 eV). These changes in the core levels can be ascribed to the increased electron density on the emitter atoms after binding to electropositive Au atoms.^17^,^55^

**Fig. 4:**
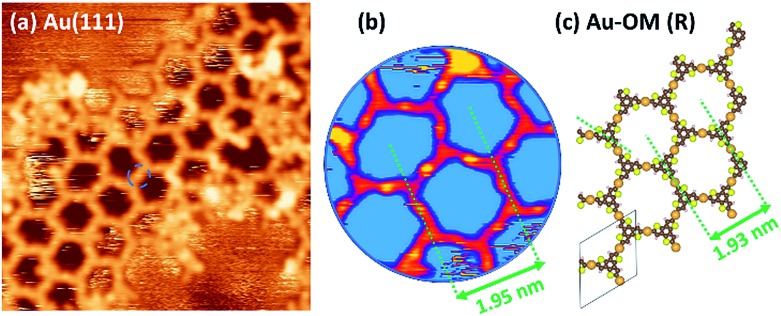
(a) 15 × 15 nm^2^ STM images of TBTTB deposited on a heated Au(111) surface kept at 200 °C (*I*_t_ = 0.23 nA; *V*_t_ = 0.97 V); (b) 3.5 × 3.5 nm^2^ zoom of (a), with different color scheme to enhance the bright spots of Au, with the average centre-to-centre distance reported (c) gas-phase DFT calculated *R*-TTB-Au OM structure. The unit cell dimensions are: *a* = 19.30 Å, *b* = 19.37 Å, with a 60° angle between *a* and *b*.

OM networks were further investigated by direct deposition on a surface held at 200 °C. When compared to the structures obtained by the annealing of the RT-deposited sample, XPS data show no chemical difference between the two cases (Fig. S4†). However, the STM images reveal striking differences, with small domains of well-ordered morphologies appearing in the heated surface (Fig. 4a).

When dosed on a heated surface (200 °C), the dehalogenated molecules do not react with their immediate neighbours but possess sufficient thermal energy and time to diffuse to optimal positions, permitting the formation of a low-density hexagonal structure. This observation is consistent with the previous work^56^ for the Ullmann reaction of dibromo-*m*-terphenyl on Cu, where the formation of macrocycles was favoured by low deposition rates.

DFT calculations were performed to simulate the observed OM hexagonal phase. The center-to-center distance measured in STM images (1.95 ± 0.10 nm, Fig. 4b) is in agreement with a calculated OM structure (1.93 nm, Fig. 4c), and is considerably larger than expected for a polymer structure (1.48 nm, Fig. S5†). The STM images also exhibit bright spots between every vertex (Fig. 4), attributed to a bridging Au atom.

Post-annealing at various temperatures up to 400 °C (or direct deposition at 300 °C) does not further improve the order of the OM phase, but instead produces a disordered phase (Fig. 2a, red frame, and S6†). The XPS spectra show a shift toward higher BE, *i.e.* 163.3 eV for S 2p_3/2_ and 284.5 eV for C 1s, consistent with the expected depletion of C–Au and formation of C–C bonds.^17^ However, the simultaneous appearance of the Au-related S 2p_3/2_ peak at 161.8 eV suggests that a fraction of the thiophene rings are open. By comparing the size of the two S 2p components, we estimate that a 10% of the thiophenes present broken C–S bonds. Moreover, there is also an overall decrease of both C 1s and S 2p peak areas as the annealing temperature increases (ESI, Section 2†).

### TBTTB on Ag(111)

**Fig. 5:**
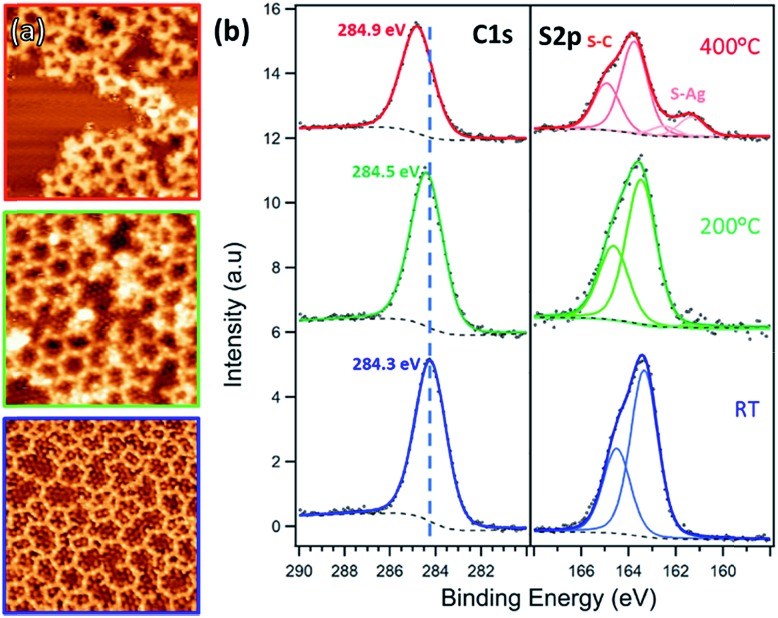
(a) STM images and (b) XPS C 1s and S 2p spectra of TBTTB deposited on Ag(111) at RT (blue, *I*_t_ = –0.48 nA; *V*_t_ = –0.11 V), and sequentially annealed to 200 °C (green, *I*_t_ = –0.30 nA; *V*_t_ = –1.40 V) and 400 °C (red, *I*_t_ = 0.30 nA; *V*_t_ = –1.16 V).

**Fig. 6:**
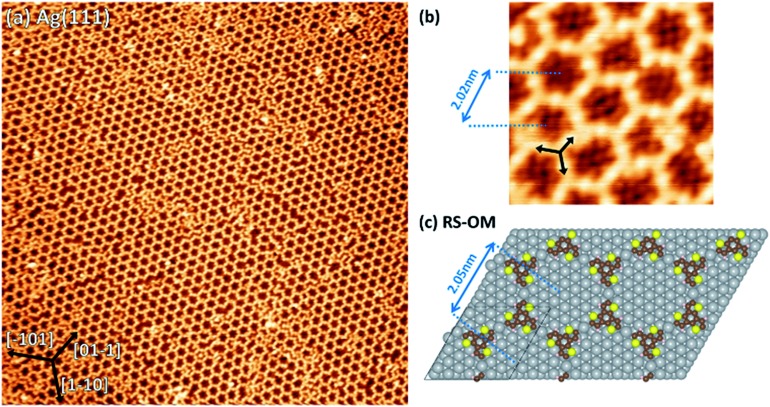
(a) 75 × 75 nm^2^ STM images of TBTTB deposited on a heated Ag(111) surface kept at 200 °C (*I*_t_ = –0.27 nA; *V*_t_ = –0.51 V). (b) a 7 × 7 nm^2^ zoom-in of (a), which exhibits the Br atoms decorating the molecular network (bright spots inside the hexagons) and the Ag adatoms between two molecules (bright spots between each connected molecule), therefore identifying the network as OM (*I*_t_ = –0.27 nA; *V*_t_ = –0.51 V). (c) DFT optimized molecular structures for OM phase of TBTTB consisting of *S* and *R* enantiomer. Silver atoms are in grey, carbon in brown, sulphur in yellow and hydrogen in light pink.

Deposition of TBTTB on silver at RT immediately yields an OM network (Fig. 5a, blue frame) composed of both open and closed polygons, with 4 to 8 vertices and irregular shapes. The order of the molecular phase increases with annealing until it forms a hexagonal closed structure above 200 °C (Fig. 5a, green frame). This suggests that the dynamic nature of the C–Ag bond is an essential feature for the network's self-assembly and “self-repair”,^57^,^58^ whereas this was not observed on gold, possibly because of the higher bond dissociation energy of the C–Au bond (1.39 eV for C–Ag *vs.* 1.99 eV for C–Au).^47^ This ordering does not occur in experiments starting at saturated coverage with subsequent annealing, presumably because molecular diffusion is hindered at higher densities. STM images at submolecular resolution reveal a random distribution of the *R* and *S* enantiomers in the porous OM networks (Fig. S10†). This difference with the close-packed self-assembled molecular networks on Au(111) is consistent with the variation in chiral expression between close-packed and porous networks observed for the related TTB-tricarboxylic acid.^59^ The Ag OM phase (Fig. 5a, green frame and Fig. 6) is commensurate with the Ag(111) substrate with an overlayer matrix of 
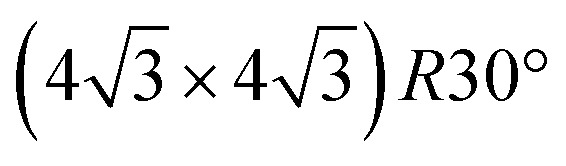
 containing two TBTTB molecules in the unit cell (Fig. S11†).

The experimental pore-to-pore distance for a hexagonal network is measured to be 2.02 ± 0.10 nm, in agreement with the calculated 2.05 nm distance of OM structure (Fig. 6b and c), calculated by first optimizing the TBTTB adsorption position on the Ag(111) surface and then building the OM structure, also including the adsorbed Br atoms (ESI Section 4†).

Annealing above 300 °C destroys the hexagonal structure, yielding a disordered network of distorted polygons (Fig. 5a, red frame) similar to the phase observed on gold. The 1.20 ± 0.10 nm vertex-to-vertex distance of the OM phase is reduced to 0.90 ± 0.10 nm after annealing to 400 °C (Fig. S15†), consistent with the transformation of the C–Ag to C–C bonds (Fig. S5†). XPS shows that the C 1s peak shifts towards higher BE, as expected for the conversion from OM to polymer phase. The appearance of a second S 2p doublet at lower binding energies (BE) is attributed to the thiophene ring opening,^18^*via* breaking a C–S–C bond and formation of C–S–Ag bonds. This suggests that the polymerization reaction competes with desulphurization, which explains the lack of order in the polymer phase. From an analysis of the S 2p peak components we can observe that at complete polymerization, *i.e.* 400 °C, 18% of the C–S bonds are broken (ESI, Section 2†). Once again, we observe a decrease in the C 1s and S 2p peak intensities during annealing (between 200 °C and 400 °C), confirming that in addition to desulphurization, the molecular fragments desorb from the surface.

To improve the domain size of the OM phase for such applications, we deposited TBTTB on a Ag(111) surface kept at 200 °C, which resulted in larger (>30 nm) hexagonally packed domains (Fig. 6a). XPS shows no difference between deposition at 200 °C and RT deposition with post-annealing to the same temperature (Fig. S4†). These hexagonal domains obtained *via* hot-surface deposition still contain multiple line defects but exhibit much higher degree of order compared to the OM networks obtained at RT.

**Fig. 7:**
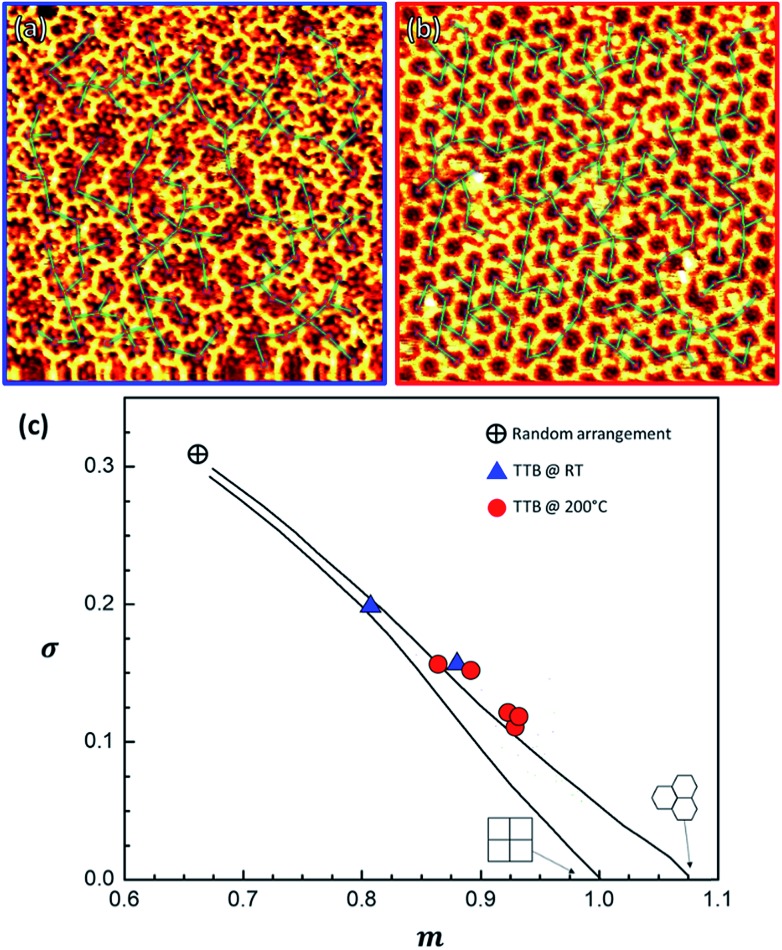
STM images of a 20 × 20 nm RT OM structure (a) and a 30 × 30 hot-dosed OM layer (b), with the MST network superimposed. (c) Plot confronting the area-normalized average of the distribution of edge lengths in the MST, *m*, *vs.* its standard deviation, *σ*, as obtained from the STM images of TBTTB/Ag at RT (in blue, *I*_t_ = 0.35 nA; *V*_t_ = 0.52 V) and hot-dosed (in red, *I*_t_ = –0.49 nA; *V*_t_ = –0.11 V); the lines show the distortion trajectory^57^ for squared or hexagonal lattice; additional information in the ESI, Section 6.†

We performed a statistical analysis of the OM lattice layout, by a minimum spanning tree (MST) network that connects the centres of mass of each closed pore (Fig. 7a and b and S16†) following the procedure previously followed by Ourdjini *et al.*^60^ By comparing the MST's statistically aggregated values (details in ESI, Section 6†) with the calculated trajectory^61^ that joins the perfect hexagonal lattice with the random arrangement point, it is evident that when TBTTB was dosed on a hot surface much more ordered structures were produced (Fig. 7c).

The OM grown on Ag is more extended than the OM obtained on Au(111). This is similar to the observations by Bieri *et al.*,^62^ who reported that high diffusivity of molecules and a low coupling probability are essential for achieving long-range ordered structures.

### TBTTB on Cu(111)

**Fig. 8:**
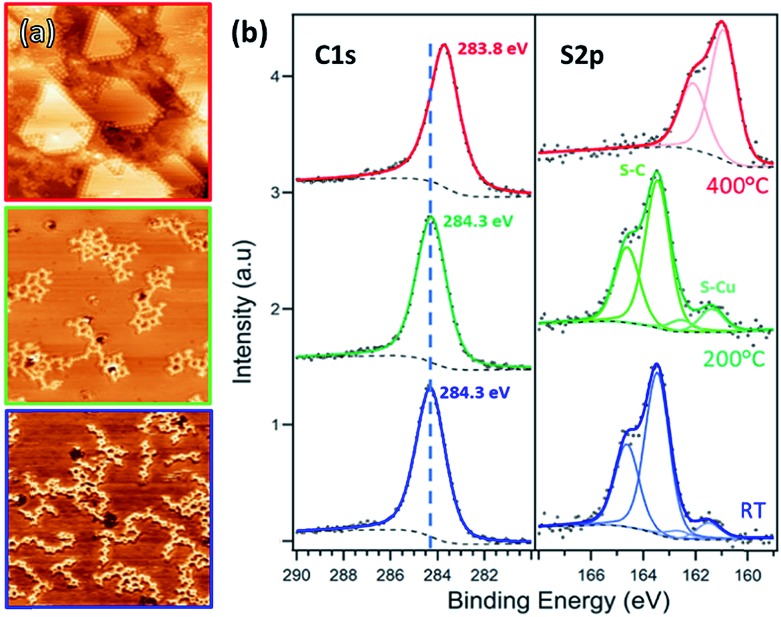
(a) STM images and (b) XPS C 1s and S 2p spectra of TBTTB deposited on Cu(111) at RT (blue, *I*_t_ = –1.02 nA; *V*_t_ = –1.50 V), and sequentially annealed to 200 °C (green, *I*_t_ = –0.53 nA; *V*_t_ = –0.97 V) and 400 °C (red, *I*_t_ = –1.45 nA; *V*_t_ = –0.98 V).

Deposition of TBTTB on copper at RT results in branched OM chains on the surface (Fig. 8a, blue frame). XPS analysis shows that the molecules are fully dehalogenated, and the C 1s peak position at 284.3 eV suggests the formation of the C–Cu bonds.^17^ The observed OM chains are similar to those reported by Bieri *et al.*^62^ for cyclohexa-*m*-phenylene on Cu(111). 2D networks were observed on gold and silver instead of the copper chain structure, presumably due to the faster diffusion of the molecules on the former substrates.^29^

Annealing up to 200 °C increases the size of the molecular domains and the number of closed structures, but no long-range order is achieved (Fig. 8a, green frame). Further annealing above 200 °C does not improve the order. Instead, the STM contrast suggests fragmentation of the molecules (Fig. 8a, red frame).

This is confirmed by the shift of S 2p_3/2_ peaks from 163.34 to 161.08 eV, characteristic of the copper-bonded sulphur, which is present even at RT, where 10% of the thiophene rings are broken (ESI, Section 2†). This percentage increases with annealing temperature, and at 400 °C only S–Au is observed, meaning that all of the thiophene rings are open. At this temperature the sulphur atoms appear to be completely removed from the molecule, both decorating the step edges and forming a distinct 2 × 2 overlayer (Fig. 8a, red frame).^63^ No long-range ordered structure was observed upon dosing TBTTB on a hot Cu(111) surface.

### Surface comparison

The choice between the three coinage metals strongly affects the final result of TBTTB deposition. At RT we observe intact molecules on Au, partially dehalogenated on Ag and fully dehalogenated on Cu. This trend fits with the halogen affinity of the three surfaces: bond dissociation energies (BDEs) for Cu–Br, Ag–Br and Au–Br bonds are 3.43, 2.90 and 2.21 eV respectively.^47^ The reported BDE values for Cu–S, Ag–S and Au–S bonds are 2.85, 2.25 and 2.63 eV respectively.^47^ While the largest BDE of Cu–S bond is in line with the fastest thiophene ring opening on this surface, the second highest BDE belongs to Au–S bond, which does not explain the lowest reactivity observed on this surface. The difference between Ag(111) and Au(111) – the two apparently similar surfaces (with almost the same nearest-neighbor distance, 2.88 and 2.89 Å respectively), is well known and has been reported for the chemically related self-assembled monolayers of alkanethiols.^64^

**Fig. 9:**
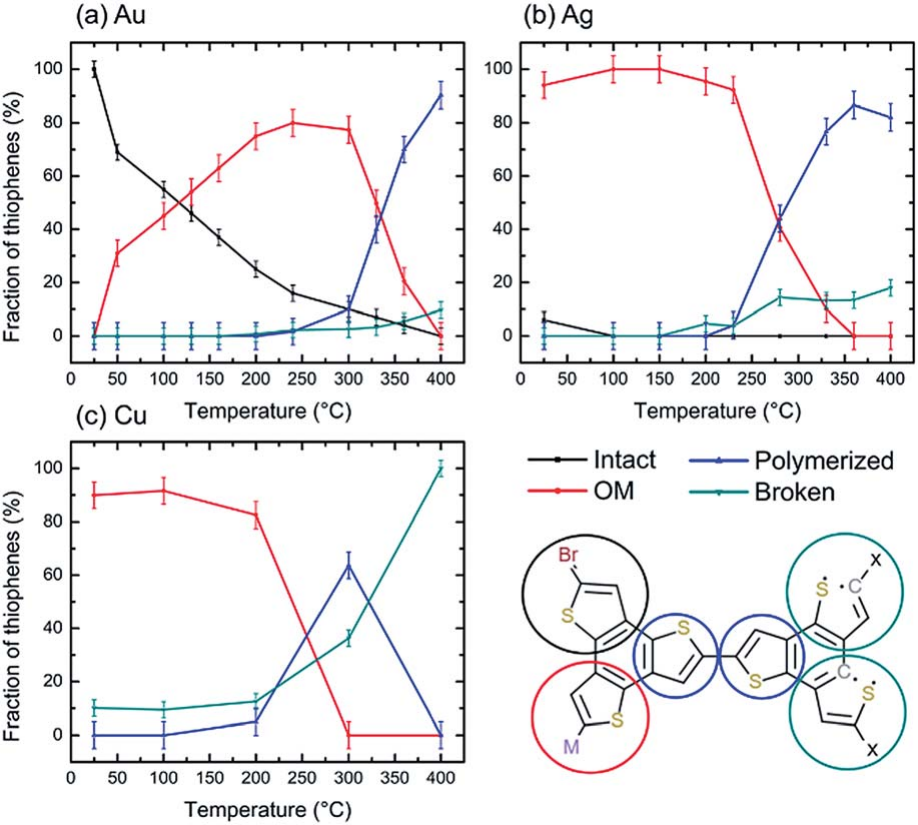
Fraction of thiophenes in each observed chemical state for the three studied surfaces at each temperature. The values are taken from the spectral decomposition of the C 1s, S 2p and Br 3p peaks, and do not consider molecular desorption.

The high chemical ‘nobility’ of Au(111) compared to all other metals is almost universally accepted, and is generally attributed to the high cohesive energy of the Au 5d states.^65^ Although thermal annealing does lead to organometallic and polymeric domains on all three substrates, the nature of the surface is still decisive in determining the purity/phase composition, as well as the molecular structure of the polymer. This is evident from the comparison in Fig. 9, which shows the fraction of monomer in each state: intact, OM, polymer, broken. While for Ag it is possible to obtain a full OM layer stable in a wide temperature range (RT-200 °C), for Au these structures coexist with both intact and polymeric moieties. This is further reflected by the lack of long-range order in the TBTTB/Au annealed phase.

For the C–C coupling polymerization, the Au substrate was the only one resulting in reasonable reaction efficiency (90% at 400 °C), but still the defects associated with the thiophene ring opening prevented the formation of ordered 2D polymers networks.

## Conclusions and perspectives

The interactions between molecules and surfaces play a key role in steering many chemical phenomena. Our investigation of TBTTB deciphers the role of these interactions in four interweaved processes: (i) molecular self-assembly, (ii) carbon–halogen bond cleavage forming OM structures, (iii) carbon–carbon bond formation producing covalent polymers and (iv) carbon–sulfur bond cleavage which open the thiophene rings in the polymer. Deposition of the TBTTB monomer at RT resulted in non-covalent molecular networks on Au(111), OM structures on Ag(111) and partially broken molecules on Cu(111).

We demonstrate that varying the substrate temperature during the deposition drives the growth process toward different final products. By depositing TBTTB on a hot surface, we formed an ordered 2D OM network on Au and a highly extended version of the same network on Ag. Further heating of the OM phase (>200 °C on Cu, >250 °C on Ag, >300 °C on Au) triggers C–C coupling of the TTB core, but the polymerization cannot be completed because of concomitant C–S bond cleavage. The maximum efficiency of the C–C coupling peaks at *ca.* 90% for Au at 400 °C (Fig. 9, ESI Section 2†). Comparison with other thiophene-containing monomers suggests that the probability of side-reactions correlates with the S/C ratio, as expected from the statistical probability of desulphurization. Thus, in the case of tetrathienoanthracene (S/C = 0.182) small polymer domains could be prepared on Ag,^18^ while for TTB (S/C = 0.25), even on the least reactive Au(111) surface, desulfurization is observed simultaneously with the polymerization. In addition, the non-fused thiophene rings might be more resistant to C–S scission due to a more significant aromatic stabilization, as observed for 3,4-(ethylenedioxy)thiophene on Ag.^37^

Despite their lower prominence in the field of soluble (1D) conjugated polymers, O– and N– containing building blocks (furan, pyrrole, pyridine, *etc.*) appear to present better alternatives for the design of surface-templated semiconducting materials, although ring opening reactions are still a problem when using highly reactive surfaces such as Cu.^66^

On the other hand, this study marks the formation of long-range ordered OM phases, covering the whole surface. Such networks can be prepared at lower annealing temperature, where no desorption and ring opening take place. The reaction efficiency on Ag is close to 100%, allowing to obtain extended domains larger than 20 × 20 nm^2^ – a size comparable with the typical feature sizes of state-of-the-art silicon technology. Due to their high degree of order and promising electronic properties, such OM structures may find different applications in molecular recognition, 2D nanopatterning and non-linear optics.^67^,^68^ The realization of these networks on the air-stable Au surface is a promising result, which opens new possibilities for their characterization outside the ultra-high vacuum (UHV), as well as a starting point for the development of OM-based devices.

## Experimental

All the experiments were performed within UHV chambers with base pressures below 2 × 10^–10^ mbar. TBTTB was synthesized as described in previous work^46^ and deposited onto the samples using Knudsen-type effusion cells at temperatures between 100–120 °C. The deposition was performed on samples held at various temperatures: RT, 150, 200 and 250 °C. The 111 single crystals of Au, Ag and Cu (MaTecK GmbH) were cleaned by repeated cycles of Ar^+^ sputtering and annealed at 450–500 °C. STM images were acquired in constant-current mode using a SPECS Aarhus 150 STM and an Omicron VT-STM. STM images were analyzed using the free WSxM software,^69^ and were treated for plane subtraction, line-by-line flattening, contrast, and corrected based on the lattice parameters of the metal substrate.

XPS experiments were performed using an electron spectrometer (SPHERA II U5 analyzer-Oxford Instruments Omicron Nanoscience), coupled to the UHV chamber that also hosts the Omicron VT-STM. The electron spectrometer consists of a hemispherical analyzer and a five-channeltron detector. All XPS analyses were performed in normal emission, using a twin anode Mg/Al X-ray source (DAR 400), supplying non-monochromatic Mg K_α_ radiation at 1254.6 eV photon energy. The BE scale of the XPS spectra was calibrated to the Ag 3d 5/2, Au 4f 7/2 and Cu 2p 3/2 photoelectron core levels at 368.25 eV, 84.00 eV, and 932.67 eV, respectively. Spectral peak fitting was based on residual minimization with Voigt line shapes and Shirley backgrounds, unless stated otherwise, and was performed using the Casa XPS software.^70^

Theoretical calculations were performed with the Vienna Ab-initio Simulation Package (VASP).^71^,^72^ DFT calculations were made using the Pedrew–Burke–Ernzerhof^73^ generalized-gradient approximation (PBE-GGA) for exchange–correlation potential, the projector augmented wave (PAW) method,^74^,^75^ and a plane-wave basis set with an energy cut off of 450 eV. The zero-damping DFT-D3 method of Grimme,^76^ was used for dispersion correction. The lattice constant of silver was first optimized (4.1362 Å) using 146-irreducible *k*-points (13 × 13 × 1 *k*-mesh), which was then used to construct the subsequent Ag(111) supercells with an energy convergence criterion of 5 meV using 61-irreducible *k*-points (11 × 11 × 1 *k*-mesh). The experimentally determined unit-cell of the adsorbed molecules on the Ag(111) surface and the epitaxy of the molecules with respect to the surface (see ESI, Section 4†) were used to construct a 5-layer slab with a vacuum gap of 15 Å. The bottom bi-layer was kept frozen and all the other surface atoms and the adsorbed molecules were fully relaxed until the net force on each atom was less than 0.05 eV Å^–1^. The Ag(111) slab constructed for calculating the OM phase contained 240 Ag atoms, arranged in five layers, with dimensions *a* = 20.47 Å, *b* = 20.47 Å, *c* = 30.00 Å, and an angle of 60° between *a* and *b* vectors (which corresponds to a (7 × 7) unit cell, as described in Section 4.1 of ESI†). All calculations for the adsorption of the molecules or their moieties on the surface were performed by sampling the gamma point of the Brillouin zone, and without using spin-polarization. However, in the case that adatoms were included on the surface (slab containing 243 Ag atoms), the convergence and energy were also tested with spin-polarization applied. The adsorption of individual TBTTB molecules on the Ag(111) surface was simulated to identify the most energetically stable adsorption site and orientation, which was subsequently used in the OM and polymer structure calculations. To simulate the molecular self-assembly, the OM network and the polymer obtained on the Au surface, because of the complexity of herringbone surface reconstruction, the DFT calculations were instead performed in the gas-phase. Images of the simulated structures were generated using VESTA software.^77^

## Conflicts of interest

The authors state that there are no conflicts to declare.

## Supplementary Material

Supplementary information
