# Supplementary material for: Surface-mediated assembly, polymerization and degradation of thiophene-based monomers
Source: Chem Sci. 2019 Apr 16;10(19):5167–75. doi: 10.1039/c8sc05267k (PMC6526482; doi:10.1039/c8sc05267k)
Supplement: Supplementary file 1 [file SC-010-C8SC05267K-s001.pdf]

Electronic Supplementary Material (ESI) for Chemical Science.
This journal is © The Royal Society of Chemistry 2019

Supporting information

Title: Surface-mediated assembly, polymerization and degradation of thiophene-based monomers.

G. Galeotti,[a,b,†] F. De Marchi,[a,†] T. Taerum,[c] L. V. Besteiro,[a,d] M. El Garah,[a] J. Lipton-Duffin,[e] M. Ebrahimi,[a,#,*] D. F. Perepichka,[c,*] and F. Rosei[a,d,*]

a. Centre Energie, Matériaux et Télécommunications, Institut National de la Recherche Scientifique, 1650 Boulevard Lionel-Boulet, Varennes, Québec, Canada J3X 1S2.
b. Istituto di Struttura della Materia, CNR, Via Fosso del Cavaliere 100, 00133 Roma, Italy
c. Department of Chemistry, McGill University, 801 Sherbrooke Street West, Montreal, Quebec, Canada H3A 0B8.
d. Institute for Fundamental and Frontier Science, University of Electronic Science and Technology of China, Chengdu 610054, PR China.
e. Institute for Future Environments, Queensland University of Technology (QUT), 2 George Street, Brisbane, 4001 QLD, Australia.
#. Current address: Physics Department E20, Technical University of Munich, James-Franck-Str.1, D-85748 Garching, Germany

† Authors share contribution

*Corresponding authors' e-mail: maryam.ebrahimi@emt.inrs.ca; dmitrii.perepichka@mcgill.ca; rosei@emt.inrs.ca

Table of Contents

1. TBTTB on Au(111) - additional STM and DFT information 2
  1.1. Dosed @ RT 2
  1.2. Dosed @ 200 °C 5
  1.2.1. Hot-dosed vs Annealed – C1s peak 5
  1.2.2. Organometallic *vs* polymer network size 5
  1.3. Dosed @ 300°C 6
2. Additional XPS data 7
3. OM network chirality 7
4. Additional details on DFT calculations 10
  4.1. STM unit cells for DFT calculation 10
  4.2. Adsorption geometry of single TBTTB molecule on Ag(111) 10
  4.3. DFT calculation including bromine atoms 11
  4.4. DFT calculation of TBTTB polymers on Ag(111) 12
5. TBTTB on Ag(111) - OM *vs* polymer dimensions 13
6. Study of order 13
  7. XPS additional data and temperature mapping of the chemical state of TBTTB 15
8. High resolution version of Figures 2 – 5 – 8 from main text 19

9. Bibliography 22

1. TBTTB on Au(111) - additional STM and DFT information

1.1. Dosed @ RT

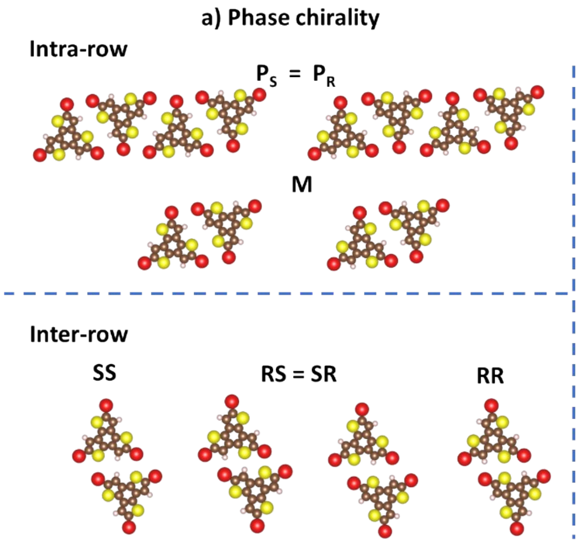

b) Phase interactions

Structure	E_{tot} (eV)	ΔE/unit cell (eV)
P_XP_X	-543.060	-1.209
P_XP_Y	-543.065	-1.214
P_XM	-543.078	-1.227
MM	**-543.105**	**-1.254**

Figure S1. a) Different combinations of R and S enantiomers in the unit cell were calculated. Two main classes of interactions can be identified: intra- and inter-rows. The figure highlights the equivalent interactions b) The total energy per unit cell and cohesive energy per unit cell are given for the calculated structures reported in Figure 1 of the main text... P_X and P_Y refers to pure (S or R) enantiomeric rows, while M refers to mixed racemic rows.

When dosed on Au(111) at RT, TBTTB molecules arrange in a long-range ordered self-assembled structure, reported here in Figure S2a, and Figure 1 and Figure 2a-blue panel of the MS. The enantiomer identification of the molecules can be obtained by assigning the chiral character to every molecule on the basis of its appearance in STM images, as shown in Figure S2a, which allow us to identify an ordered racemic mixture of the elf-assembled layer. To confirm this point we performed gas-phase DFT calculations as discussed in the main text, which show that the racemic mixtures are more stable, while pure enantiomeric ones are more energetic. In Figure S1 we identify all the possible interactions. By examining their symmetry, we can reduce the number of possible intra-row configurations to two, one relative to an enantiopure row, labeled P_S or P_R depending of the enantiomer, and the other relative to racemic composition M (Figure S1a). The possible inter-row interactions depend on the composition of the rows and the shift between them (Figure S1a).

Due to symmetry considerations, there are thus four non-degenerate configurations for the self-assembled structure of TBTTB, based on the composition of the single rows. In Figure S1b, we report the highest cohesive energy values for each of the calculated combinations, showing that the phases with enantiopure

rows are the less stable. In addition to being more stable, the MM phase also matches the enantiomer recognition from the STM images, hence this structure is reported in Figure 1 of the MS.

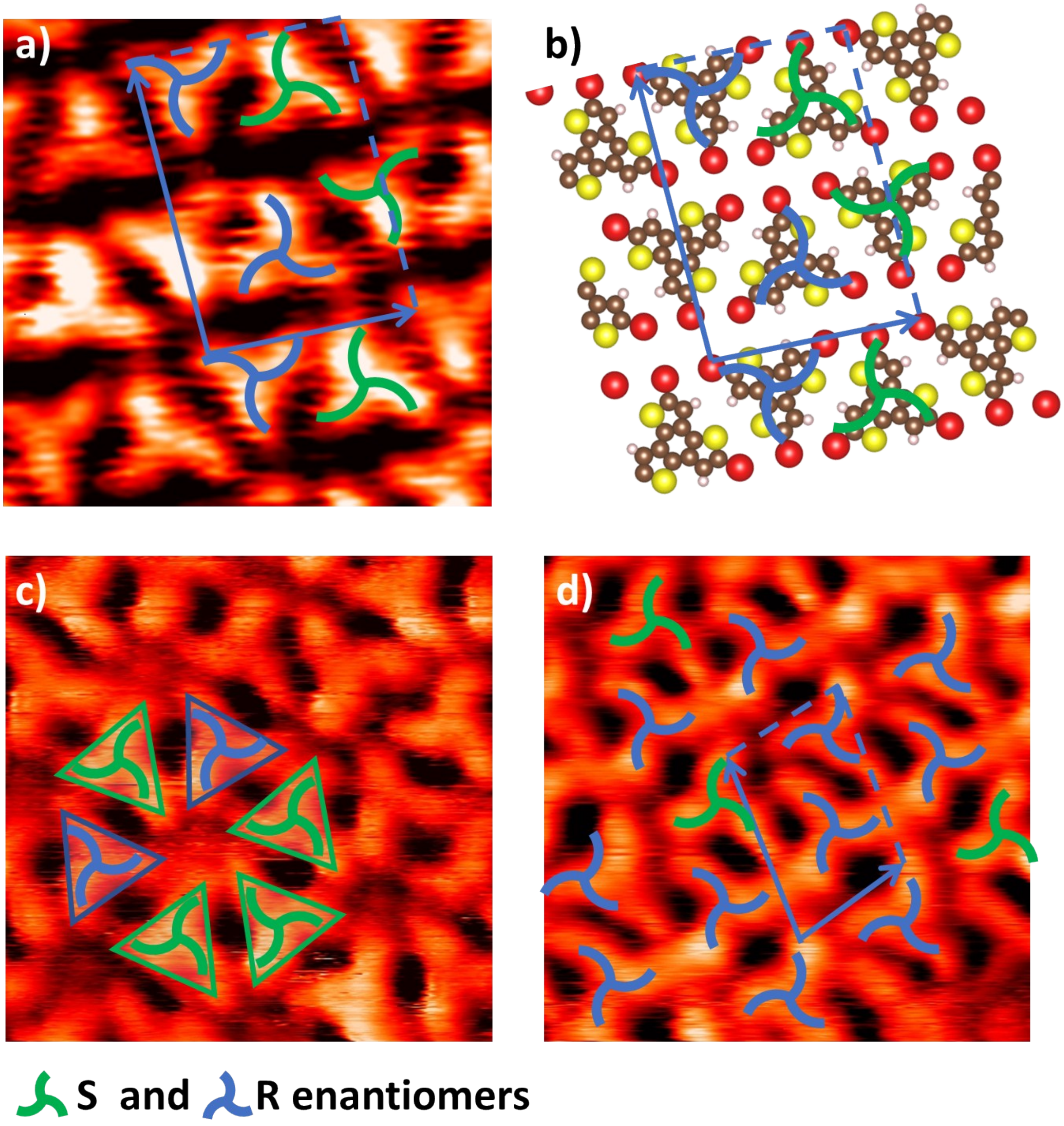

Figure S2. Close-packed self-assembly of TBTTB on Au(111) at RT, a comparison between STM image (a, 4 × 4nm²) and DFT calculated structure (b). The 4.5×4.5 nm² image of 1 ML TBTTB deposited at RT (c) and annealed at 120 °C (d). The original structure is partially destroyed, and patches of hexagonal (b) or linear (c) domains are found on the surface; annealing above 200 °C destroys the ordered phase resulting in a disordered network.

Annealing the RT phase reduces the long-range ordering of the molecules, and above 100 °C two short-range repeating structures (shown in Figure S2c-d) are observed coexisting with the RT phase. XPS measurements show that at 100 °C the Br-C bonds start to dissociate, as can be inferred from the new Br 3p doublet peak emerging at 181.4 eV and 188.0 eV, attributed to Br-Au bonds.

The appearance of the Br-Au peak proceeds in conjunction with the reduction of the Br 3p doublet peak at 183.7 eV and 190.2 eV assigned to Br-C.

This observation, together with the slight shift of C 1s (from 284.8 to 284.6 eV at 100 °C), suggests the simultaneous presence of dehalogenated molecules forming short OM chains and intact molecules, with a pattern (Figure S2d) that has changed from the close-packed structure obtained at RT (Figure S2a). The interactions between different moieties in this assembly make it possible for the molecules to rearrange into a hexagonal phase (Figure S2c), with a hexameric X6 synthon arrangement that resembles the structure observed by Gutzler *et al.* for the same molecule at the solid-liquid interface on HOPG.[1]

Further annealing of this structure keeps reducing the order of the phase, resulting in a completely disordered phase at 200 °C, as observed in the STM images (Figure S3a, b). Started at 100 °C, the Br-C bond dissociation continues up to 200 °C, and is completed at 300 ºC, where only Br-Au bonds are observed (Figure 3 and Figure S15). At sub-monolayer coverages, the Au herringbone reconstruction is completely twisted, with the network present only on the enlarged FCC portion (Figure S3a, b). Based on previous reports,[2-5] this is due to the presence of the electronegative bromine atoms, which lifts the Au surface reconstruction, so that the Au atoms release the stress.

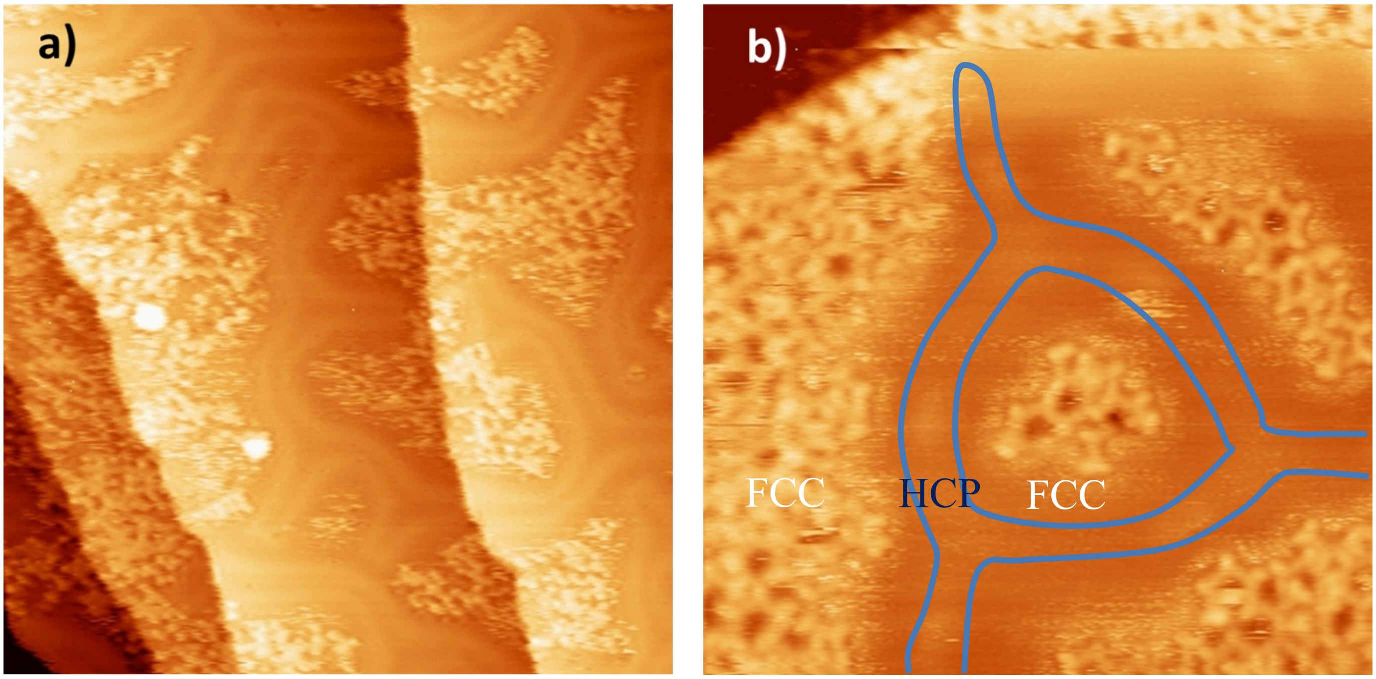

Figure S3. STM images of a submonolayer deposition of TBTTB on Au(111), dosed at RT and annealed to 150 °C. Images are 30 × 30 nm² (a) and 10 × 10 nm²(b); in both images, it is possible to see how the herringbone structure is warped and the molecular networks grow on the FCC part only.

1.2. Dosed @ 200 °C

When dosed on Au(111) at 200 °C, TBTTB forms an ordered hexagonal network, as explained in the main text. This structure is identified as an OM structure from both XPS and STM data.

1.2.1. Hot-dosed vs Annealed – C1s peak

Dosing TBTTB on pre-heated metal surfaces yield highly ordered structures with respect to dosing at RT and subsequently annealing, as discussed in the MS. While STM suggest that in both case we have OM structures, and the difference therefore relies on kinetic factors, we also used XPS to confirm our hypothesis. Figure S5 shows the comparison between the C1s peaks when hot-dosing or dosing+annealing, for Au. While the resolution is lower for the hot-dosed, due to both a lower integrating time and to a lower quantity of molecules (due to the lower density of the ordered phase), it is still possible to compare the spectra.

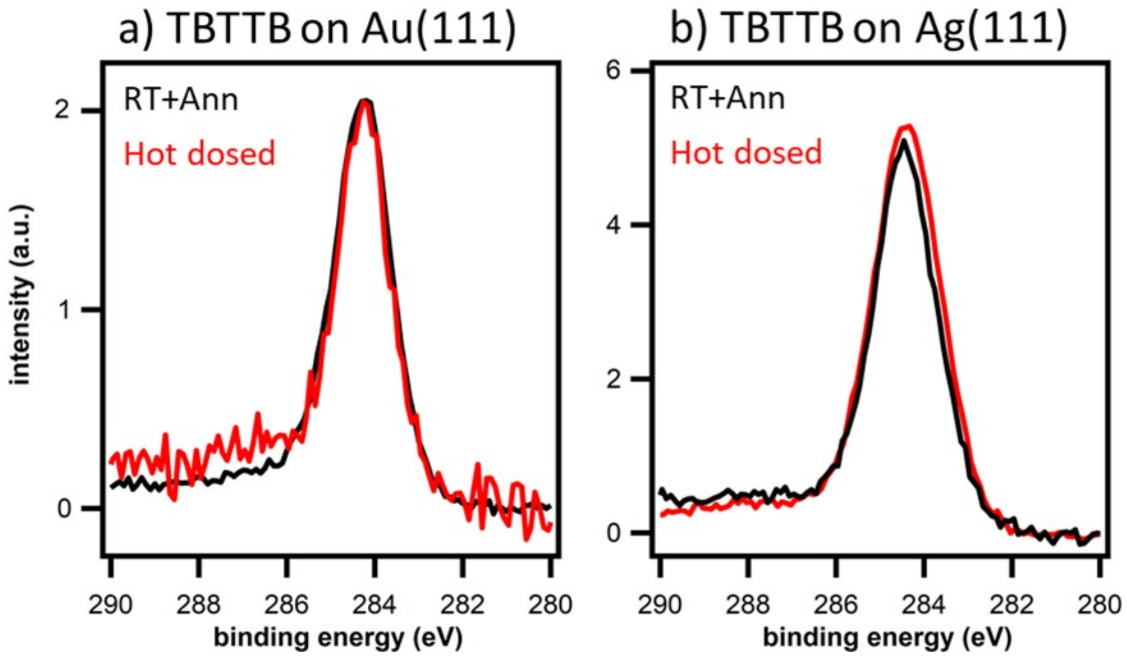

Figure S4. XPS C 1s spectra for TBTTB dosed on at RT and annealed to 200°C (black curves) and dosed on a surface kept at 200°C (red) on Au(111) (a) and Ag(111) (b). The different signal-to-noise ratio of the red curve in a is due to a shorted acquisition time, while the larger FWHM of the red curve in b is due to a higher pass energy used (20eV instead of 15eV).

1.2.2. Organometallic *vs* polymer network size

In Figure S5 the DFT calculated distances of both an OM and a polymeric structure are reported. The experimentally measured distance is equal to 1.1±0.1 nm (Figure 4c), which is in agreement with the calculated OM distance of 1.11 nm (Figure S5a).

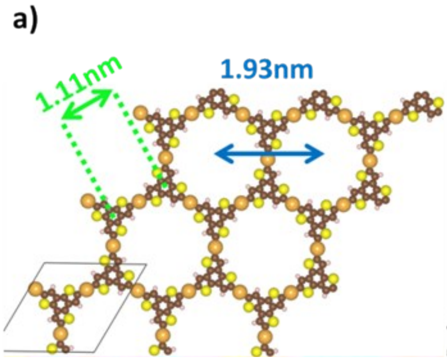

Figure S5. DFT calculation of the hexagonal organometallic structure (a) and its respective polymer (b). The dimensions are reported on the images;

1.3. Dosed @ 300°C

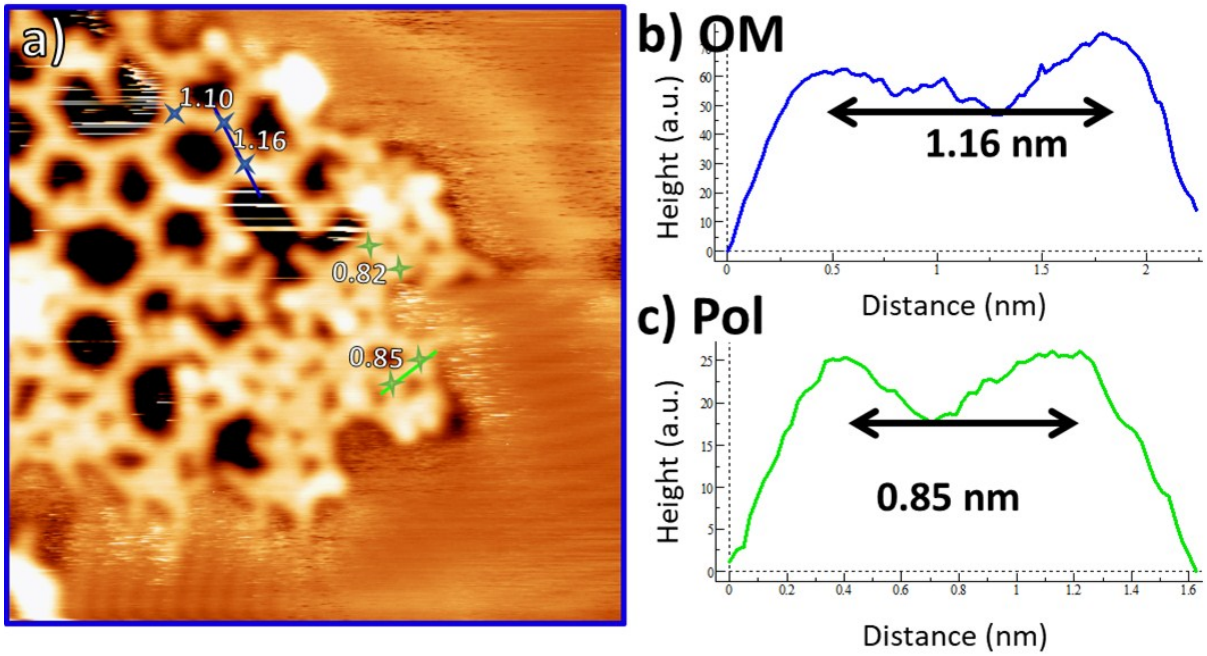

Figure S6. 15 $\times 15$ nm2 STM image of TBTTB on Au(111) at 300 °C. Both disordered OM and disordered polymers are formed on the surface upon adsorption.

2. Additional XPS data and temperature mapping of the chemical state of TBTTB

Table S1: For each sample, the element area of the XPS spectra is reported at RT and at different annealing temperatures

	Temperature °C	C 1s %	C 1s σ	S 2p %	S 2p σ	Br 3p %	Br 3p σ
Au	RT	67.4	0.8	16.6	0.6	16.0	0.6
	100	66.6	0.8	17.2	0.6	16.3	0.6
	200	69.4	1.0	17.7	0.8	13.1	0.8
	300	72.1	1.0	18.1	0.8	9.8	0.8
	400	81.4	1.4	18.6	0.8	0.0	1.6
Ag	RT	68.2	1.3	17.7	0.8	14.2	0.9
	100	68.1	2.4	17.0	1.7	14.9	2.1
	200	67.0	2.6	17.8	1.8	15.1	2.2
	300	66.2	2.0	16.8	1.1	17.0	1.6
	400	71.9	2.1	18.1	1.3	9.9	1.8
Cu	RT	73.9	1.9	12.7	1.0	13.3	1.0
	100	73.7	2.0	12.9	1.0	13.4	1.1
	200	73.6	2.4	12.5	0.7	13.9	1.9
	300	71.7	2.4	13.3	1.2	14.9	1.3
	400	81.8	1.7	13.1	1.2	5.2	0.6

When dosed on Cu, the thiophene rings of the TBTTB molecule are already partially opened at RT and only S-Cu bonds are observed at 420 °C. When deposited on Au and Ag, the molecules are intact at RT, and S-M (sulfur-metal) bonds are observed only when the surface is heated at 300 °C. While on these surfaces the degradation is small in percentage, each molecule has three thiophene rings, and therefore a mere 10% of S-M bonds can affect up to 30% of the molecules, hence drastically reducing the structural order.

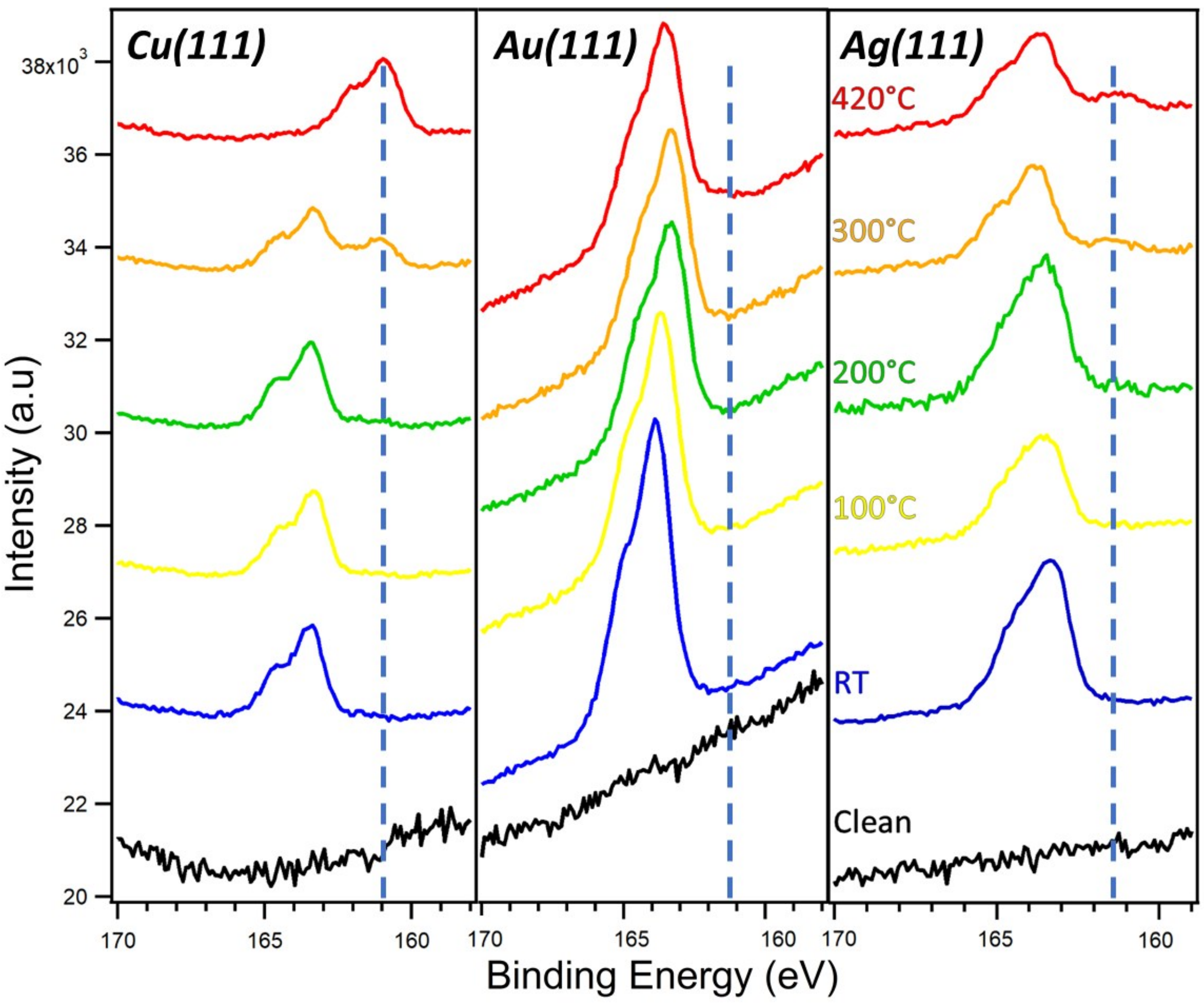

Figure S7. S 2p XPS spectra of 1 ML of TBTTB at various temperatures on three studied surfaces. Each set of plots belongs to sequential annealing of one studied sample with 20 minutes of heating at each temperature followed by XPS analysis. The dashed blue line is related to the position of the Met-S component.

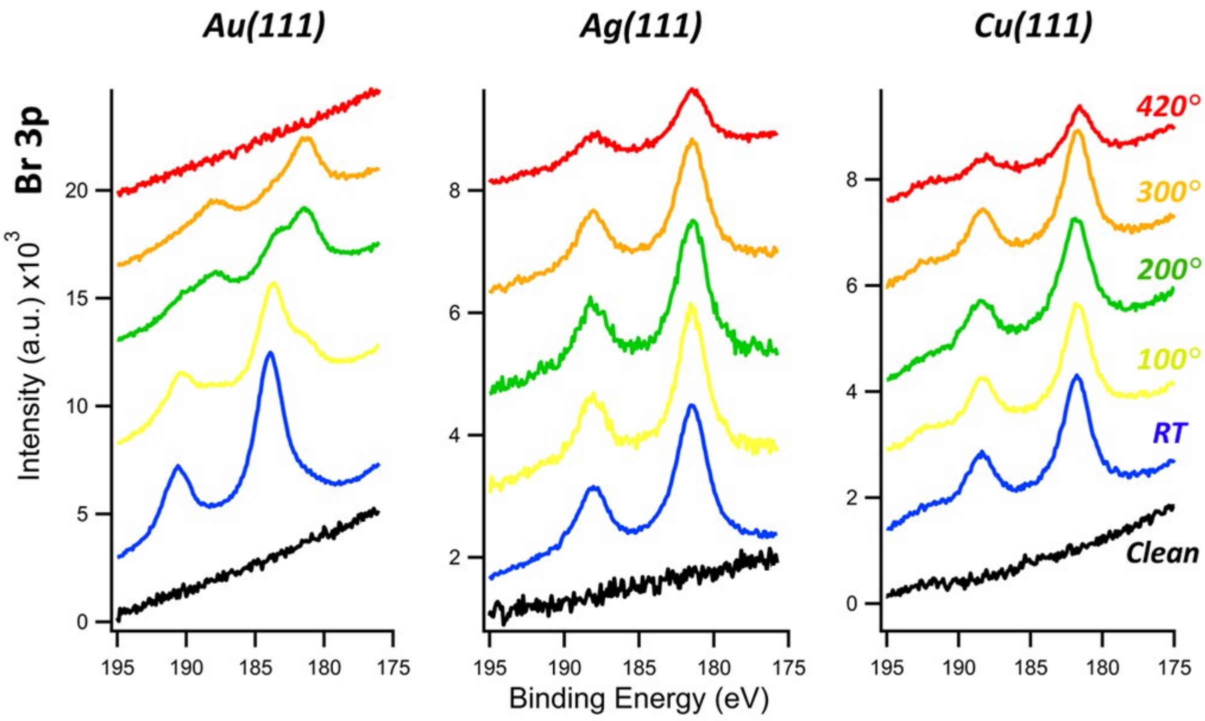

Figure S8. Br 3p XPS spectra of 1 ML of TBTTB at various temperatures on three studied surfaces. Each set of plots belongs to sequential annealing of one studied sample with 20 minutes of heating at each temperature followed by XPS analysis.

This data shows that bromine atoms undergo chemical shifts, due to making bonds with Au atoms, as described in the MS, before they entirely desorb from the Au surface. On Ag and Cu, the chemical status of bromine atoms remains the same as the adsorption upon dehalogenation, throughout the annealing steps. At 420 °C no Br atoms remain on the Au surface, while only a partial desorption is undergone on Ag and Cu (Figure S8).

Putting together all data presented in the MS and recalled here in Figures S7 and S8 we can produce a map of the chemical state of the monomer's thiophene rings at each studied temperature, which is reported in Figure 9 in the main text, in the TOC and in Table S2. However, from Figure S7 we do observe a consistent molecular desorption, and we therefore include in Figures S9 the same composition *vs* temperature map but accounting for the desorption. In both cases, from our data it is not possible to discern between a completely broken molecule and several with only one broken thiophene. Moreover, the molecules could be in a mixed state, *i.e.* one lobe could be in an OM state, another in a polymeric one. Therefore, the composition *vs* temperature maps are referred to single thiophene rings, and not to TTB monomers. The amount of intact molecules at each temperature is obtained from the percentage of de-halogenated molecules, while the amount of broken thiophenes from the percentage of S-Au. The

amount of OM and polymers instead has been obtained by analyzing the shift of the C 1s peak, and the line profile distances of the STM images, which showed the absence of polymers at RT and the absence of OM bonds at 400°C for all surfaces.

Table S2. For each sample, the monomer's thiophene rings chemical state percentage is reported at different temperatures.

	Temperature °C	Intact	OM	Polymer	Broken
Au	25	100.0	0.0	0.0	0.0
	50	69.0	31.0	0.0	0.0
	100	55.0	45.0	0.0	0.0
	130	46.0	54.0	0.0	0.0
	160	37.0	63.0	0.0	0.0
	200	25.0	75.0	0.0	0.8
	240	16.0	80.0	1,7	2.3
	300	10.0	77.4	10.0	2.6
	330	7.0	49.7	40.0	3.3
	360	4.0	20.5	70.0	5.5
	400	0.0	0.0	90.3	9.7
Ag	25	6.0	94.0	0.0	0.0
	100	0.0	100.0	0.0	0.0
	150	0.0	100.0	0.0	0.0
	200	0.0	95.4	0.0	4.6
	230	0.0	92.2	4.0	3.8
	280	0.0	40.6	44.0	14.4
	330	0.0	10.1	76.7	13.2
	360	0.0	0.0	86.6	13.4
	400	0.0	0.0	82.0	18.0
Cu	25	0.0	89.9	0.0	10.1
	100	0.0	91.6	0.0	9.4
	200	0.0	82.5	5.0	12.5
	300	0.0	0.0	63.7	36.3
	400	0.0	0.0	0.0	100.0

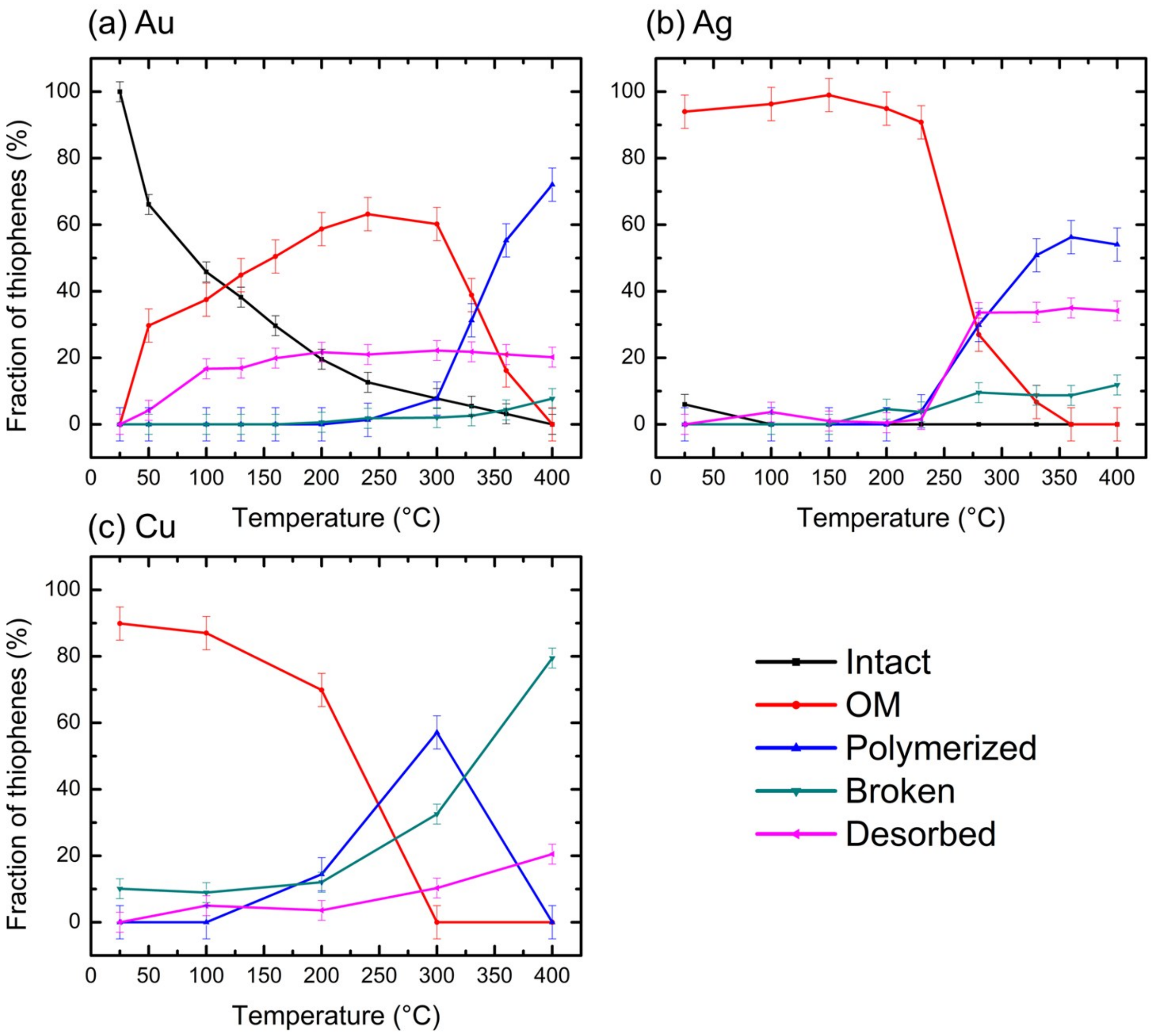

Figure S9. Fraction of monomers in each observed chemical state for the three studied surfaces at each temperature; differently from Figure 9 in the main text, these graphs take into account molecular desorption.

3. OM network chirality

The enantiomer identification of the TBTTB molecule was obtained by processing the STM images. In the case of self-assembly of intact molecules on Au, we assigned the chiral character to every molecule on the basis of its appearance in STM images. For the OM network, the molecules are dehalogenated and interact more strongly with the surface. Hence, the overall appearance in STM images can be related to the lattice site where the molecules are located; therefore, we used different procedures to identify the enantiomers. We applied three different identification methods to identify the features. An application of the three approaches on a case image is reported in Figure S8.

For the first method, we focused only on complete hexagons, on which we imposed triangles on top of every hexagon side, with the same orientation (with the base of the triangle parallel to hexagon side). We then proceeded to rotate these triangles to match the STM appearance and reported the shift as an angle. However, this method allows recognition of closed polygons only, as the open pores lack the necessary symmetry.

The second approach focuses rather on the C-Ag bonds. Every molecule is in fact bound to three silver adatoms belonging to a network. We therefore built a triangle between the Ag atoms around every molecule, then imposed a triangle on top of the molecule, again trying to match the appearance in STM. Finally, we evaluated the shift between the two triangles. However, this method allows recognition of molecules which belong to the OM networks only and does not work for single molecules.

Finally, we tried to identify the molecules by STM appearance only, as done in the Au case, and we compared the result with the other two methods. The results are quite satisfactory, and in 97% of the cases the identification was the same between the third and the first two methods. We therefore decided to use this last approach, more convenient to apply and valid for all the visible molecules.

Applying this method to all the different cases studied (TBTTB on Au and Ag at different temperatures) states that while we always observed a racemic mixture, the enantiomers where arranged in an ordered phase only in the case of intact molecules (TBTTB on Au(111) at RT), and they were randomly mixed in all the other cases (OM and polymers).

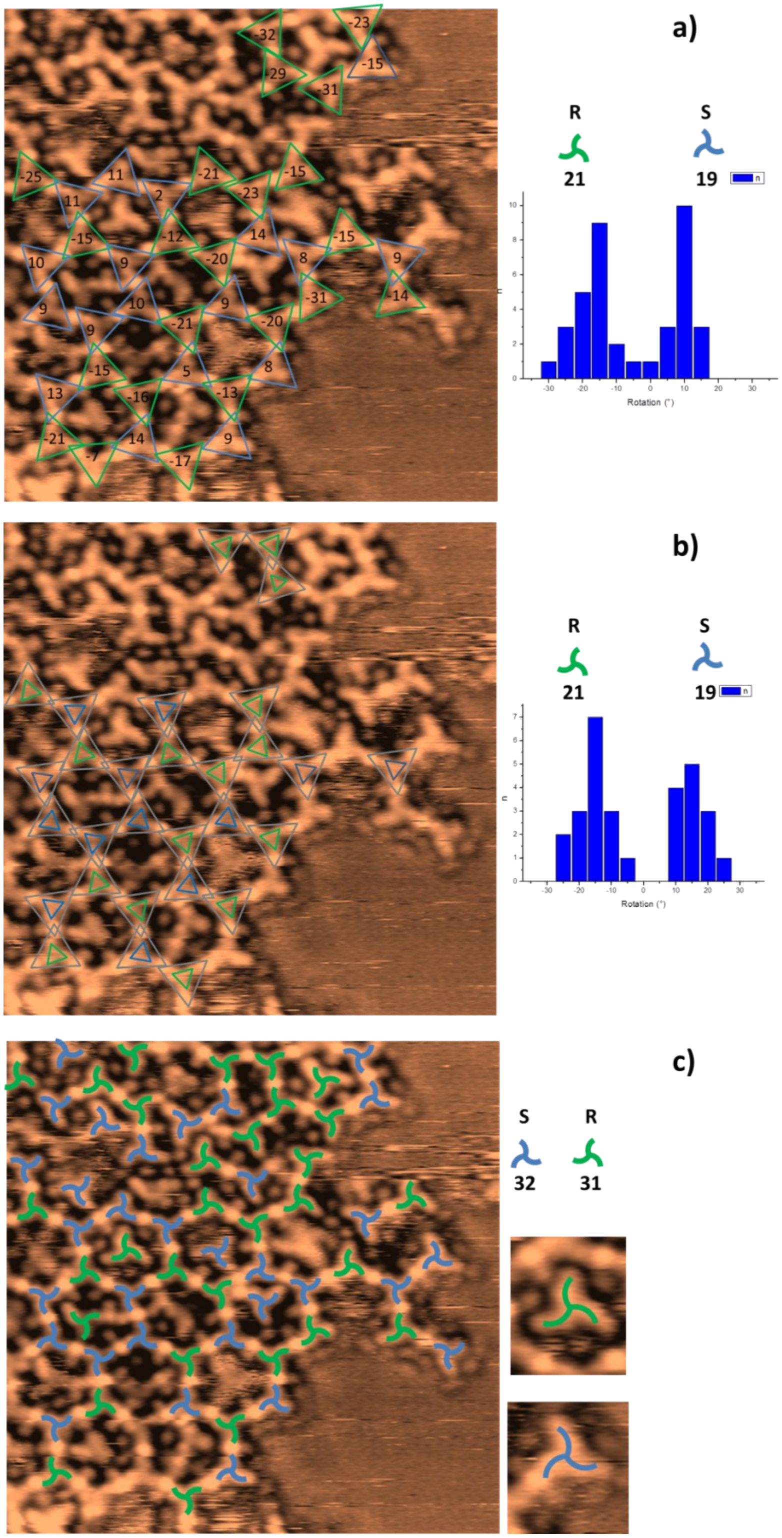

Figure S10. 15 × 15 nm^2 STM image of TBTTB on Ag(111). In all the three cases, we attempted to identify the chirality of the molecules, using different approaches described above. The graphs in a, b report the angular differences evaluated in the relative figures. In both cases, it can be noted that two peaks are present, meaning that we have a mixture of both the enantiomers. In the last case (c) two zoom-in of the image (1.5 × 1.5 nm^2) are reported.

4. Additional details on DFT calculations

4.1. STM unit cells for DFT calculation

By calibrating the images on both Ag and Au, it was possible to estimate both the pore-to-pore distance between two adjacent hexagons, and the molecule-to-molecule distance (which is taken as each side of the hexagon). Because of the intrinsic defected structure of our network (due to the presence of a racemic mixture), the dimension was measured as an average of the distance across several hexagons (results are shown in Figure S7b and Figure S8). The experimental measured distance is similar in both cases, and is equal to 2.0±0.1 nm for Ag(111), and 1.9±0.1 nm for Au(111). The DFT structure was also built starting from these analyses. The superimposition of the unit cell used for DFT simulation with an experimental image is shown in Figure S9c.

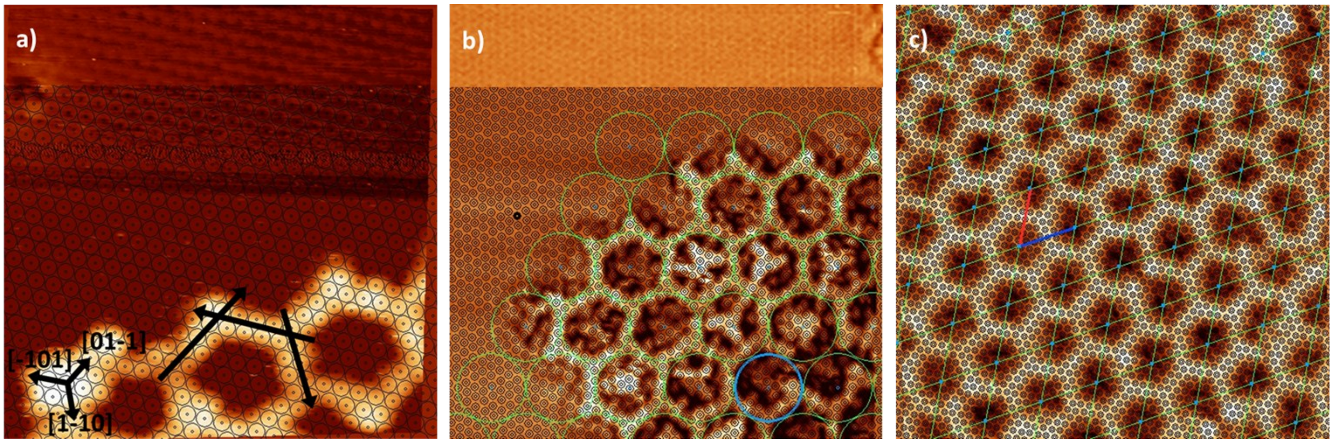

Figure S11. **6×6 nm^2 12×12 nm^2 and 16×16 nm^2 calibrated STM images of TBTTB dosed on Ag(111) kept at 200 °C. Superimposed on the STM images are the substrate lattice points (small black circles), and a) a hexagonal lattice with a 2.02 nm lattice periodicity; b) the molecular unit cell used to construct the supercell for the DFT calculations (red and blue vectors). a,b) the top part of the image shows the atomic resolution on the Ag(111) substrate, which has been used to construct the superimposed lattice point (black circles), and shows that the hexagons sides are oriented along the surface base vectors. The details and dimensions used for the DFT calculations are described in the Experimental section of the MS.**

4.2. Adsorption geometry of single TBTTB molecule on Ag(111)

DFT calculations were performed to study the interaction of the TBTTB molecule with the underlying Ag(111) surface. Different calculations with the molecule in four different adsorption positions were performed to determine the most favorable position. These positions are described with respect to the central benzene ring of the molecule: on top, FCC hollow, HCP hollow and bridge, shown in Figure S11. Both top and side view show that the molecule adsorbs in a flat conformation, and the molecular backbone is not deformed. The adsorption energy of the molecule at the HCP hollow adsorption site was

lower than other positions (more stable). To further evaluate other possible locations of the molecule with respect to the position of bromine and sulfur atoms relative to surface atoms, a few other calculations were performed by keeping the phenyl ring at HCP hollow, while rotating the molecule on plane. The most stable geometry was found to be the HCP hollow (Figure S11) in which the Br atoms are oriented along the [110], [101] and [001] vectors. The adsorption energy of this geometry is 63 meV more stable than the other simulated orientations.

This adsorption site and orientation of the TBTTB molecule was used to calculate the OM and polymer structures (Figure 6, S12 and S13).

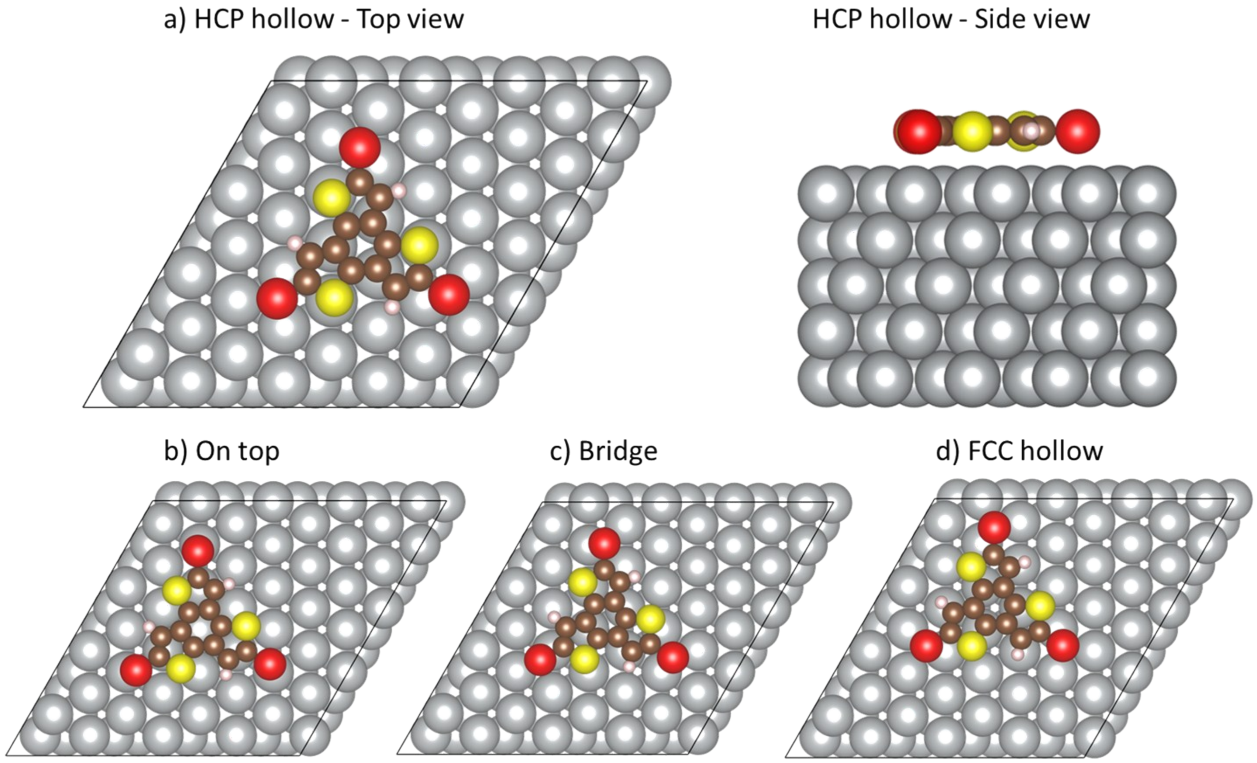

Figure S12. DFT calculated geometry of one intact molecule of TBTTB on an Ag(111) surface supercell. The molecule was located over different adsorption site, and the energies were compared upon relaxing the structure except the lower bilayer of Ag(111) was kept frozen. The top and side views of the most energetically stable structure is shown in a. dE (HCP hollow) = -2.575 eV; (on top, b) = -2.512; (bridge, c) = -2.536; (FCC hollow, d) = -2.519 eV

4.3. DFT calculation including bromine atoms

The OM structures containing the debrominated -TTB were calculated for SR (Figure S10a) and SS (Figure S10b) on a Ag(111) slab. Two supercells were chosen for Ag(111), one containing 240 atoms in which the surface atoms are pulled out the surface to incorporate in the OM structure, and another slab

having 243 atoms where three Ag adatoms are included on the surface. The method and accuracy of the calculation is described in the Experimental section in the main manuscript, and as stated for the 243 atoms slab, the data from the spin polarized calculation is reported. The adsorption site of adatom gave a slightly more stable energy (-12 meV per adatom) for the FCC compared to HCP. Nevertheless, according to the most energetically stable location of the TBTTB molecule (Figure S11a), the HCP adatoms were used to generate the OM structures.

Based on the STM experimental data, bromine atoms were identified to have remained on the surface, decorating the OM network.

Therefore, we performed DFT simulation of the OM networks by including bromine atoms to the surface. The location of bromine atoms was also optimized giving the more stable structure in FCC position than HCP (-47 meV per bromine atom for 240-Ag and -13 meV for 243-Ag slab). The OM structures on both 240 atoms and 243 atoms Ag(111) slabs in the presence and absence of bromine atoms were optimized for SS and RS TBTTB. The pure enantiomeric OM hexagons (SS-OM) were calculated to be more stable than their racemic mixture counterparts (RS-OM), as follows. On the 240-Ag slab, this difference was: $dE = E_{SS\text{-}OM} - E_{RS\text{-}OM}$= -0.310 eV (-0.315 on the brominated surface); and it was -0.324 eV (-0.386 on the brominated surface) on the 243-Ag slab (Figure S12).

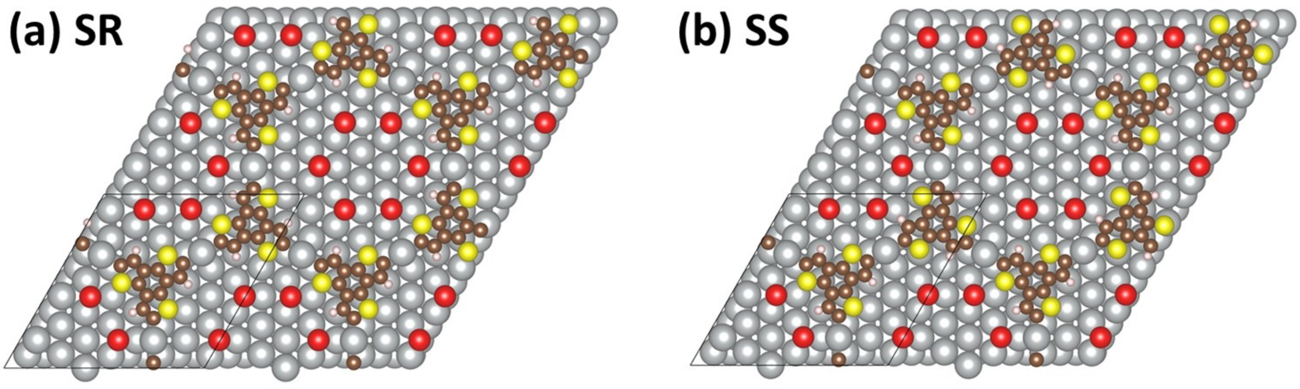

Figure S13. DFT optimized structures of TBTTB OM on Ag(111) containing adatoms. The threefold hollow adsorption site was chosen as the starting point for the Br (in Red).

4.4. DFT calculation of TBTTB polymers on Ag(111)

The 5-layer Ag(111) slab for simulating the polymer contained 135 Ag atoms, with the dimension of a=15.35 Å, b=15.35 Å, c=30.00 Å, and the angle between a and b vectors=60 degrees. The results for both polymers made by S- only or a mixture of S- and R- enantiomers are shown in Figure S13. Although

our XPS data confirms the formation of a polymer, the simulation of ordered polymer was aimed to identify the difference between the dimensions of OM hexagons from the polymeric structures.

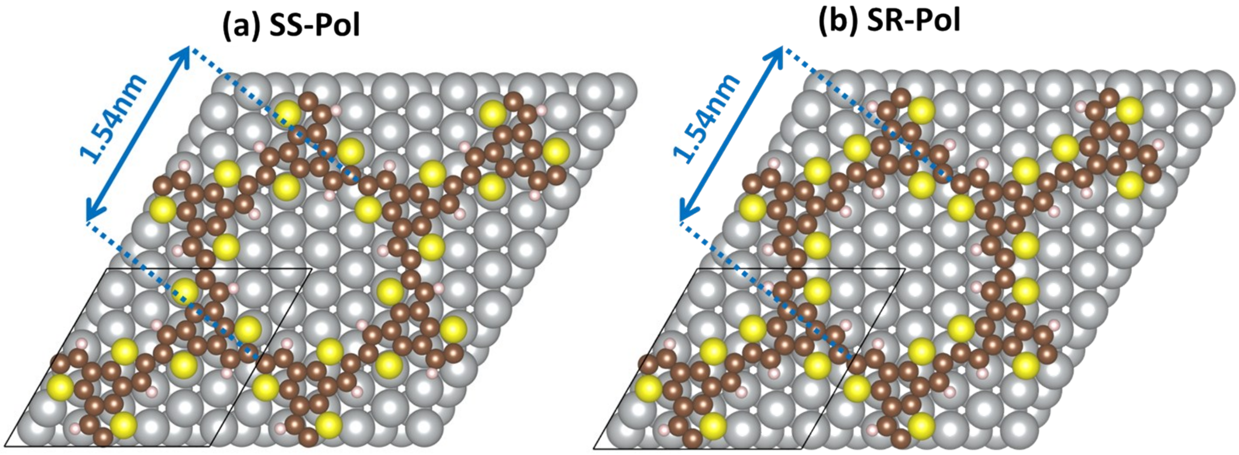

Figure S14. DFT optimized molecular structures for the imaginary ordered polymeric network of TBTTB on Ag(111), made of a) S only, and b) both S and R enantiomers. The center-to-center distance between two adjacent hexagons is reported.

5. TBTTB on Ag(111) - OM *vs* polymer dimensions

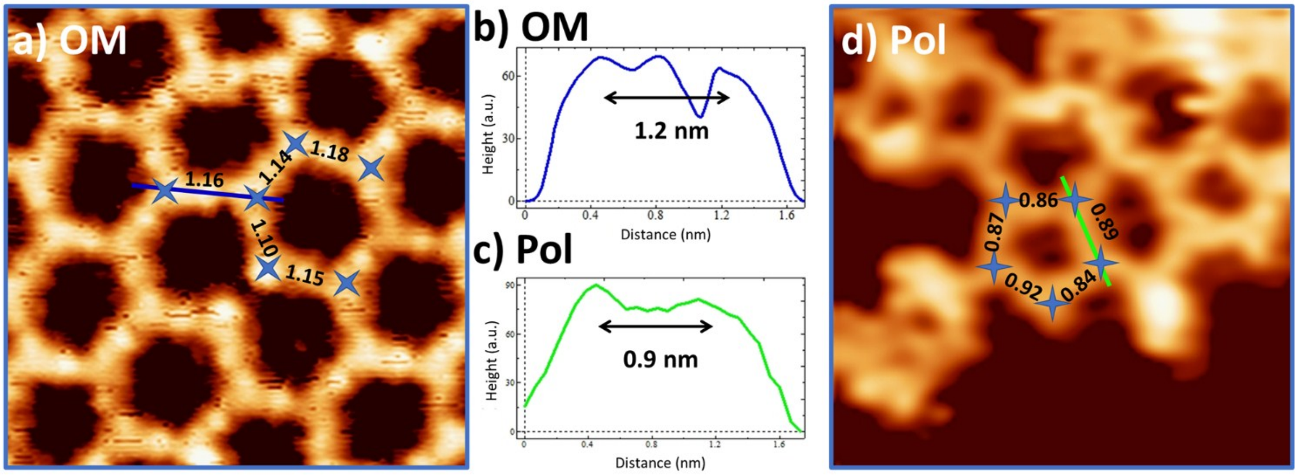

Figure S15. a) STM image of TBTTB dosed on Ag(111) at 200 °C. Blue line represents the STM line profile, shown in b; various distances between molecules are reported; b) line profile of the blue line in (a). c) line profile of the green line in (d). The reported distances in (b) and (c) are the averages from various profiles; d) STM image of TBTTB dosed on Ag(111) at 300 °C. Green line represents an STM line profile, shown in c; various distances between molecules are reported.

6. Study of order

STM images can show the overall appearance of the obtained phases, yet they do not yield a measure of the order of each phase. While we can easily infer (by visual inspection) that the hot dosed phase shows a more ordered pattern than the RT one, we wanted to use a standardized way to measure the deviation

with respect to a perfect hexagonal structure, to quantify the improvement, if any, arising from the procedure.

To this end, we used the procedure successfully exploited by Ourdjini et al.[7] In their work, they statistically analyzed the network properties of the minimum spanning tree (MST) of the network created by taking the closed cells in the surface as its nodes. This procedure allows to compare the tessellation generated by the 2D polymer with respect to perfectly ordered networks with different primitive geometries. To obtain these statistical data, it is first necessary to process the STM images to detect the different regions enclosed by the molecules, *i.e.* the different "cells", and identify their centers. As the next step, we constructed an undirected network, determining the neighbours of each node with the aid of a Voronoi tessellation process. As it can be seen in Figure S16(b), we did not include in the network the cells contiguous to the image borders, to avoid the undesired bias that the framing would introduce. Finally, the MST, which will be the subject of the statistical analysis, is found.

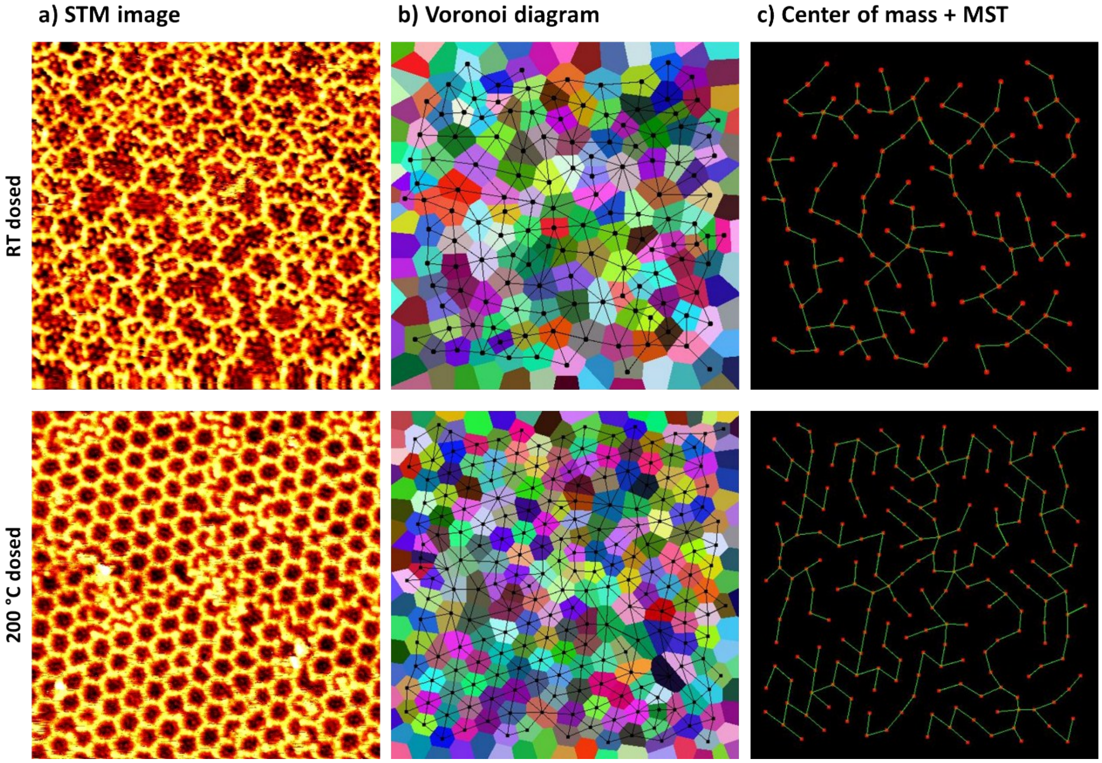

Figure S16. a) STM images of TBTTB on Ag(111), with the undirected network connecting the centers of each cell and Voronoi tessellation (b) and minimum spanning tree (c).

We seek to extract the area-normalized average (m) and standard deviation (σ) of the distribution of edge lengths in the MST, as it has been shown that these two values can characterize a pattern in terms of their geometrical primitives (*i.e.* squared, hexagonal, etc.) and its degree of order.[8, 9] Figure S17 shows the data for our systems, together with data from Ourdjini et al.,[7] where one can see the comparatively high order of our organometallic polymers in a quantitative way, as well as how the hot dosed system offers a higher degree of order.

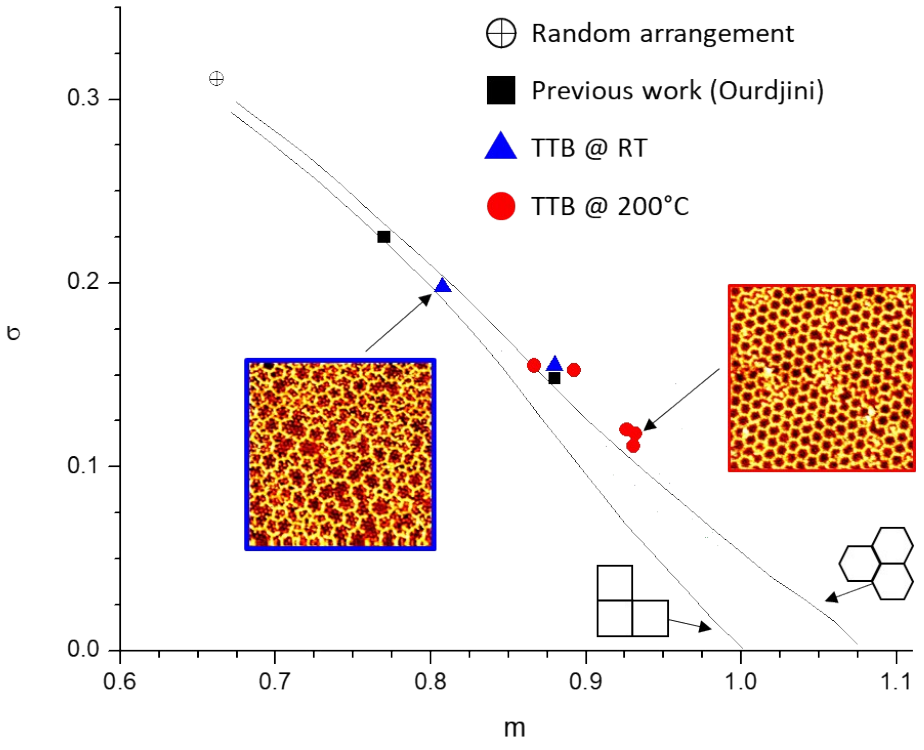

Figure S17. ***m*** **(average) vs** ***σ*** **(standard deviation) plot of the edge lengths in the MST constructed from the STM images. The position of the points relatively to the random arrangement and perfect hexagonal or square structure reflects the overall quality of the polymer. In black we reported the data from literature, in blue the best and worst samples of RT dosed TBTTB and in red the best and worst samples for TBTTB dosed @ 200°C.**

7. High resolution version of Figures 2 – 5 – 8 from main text

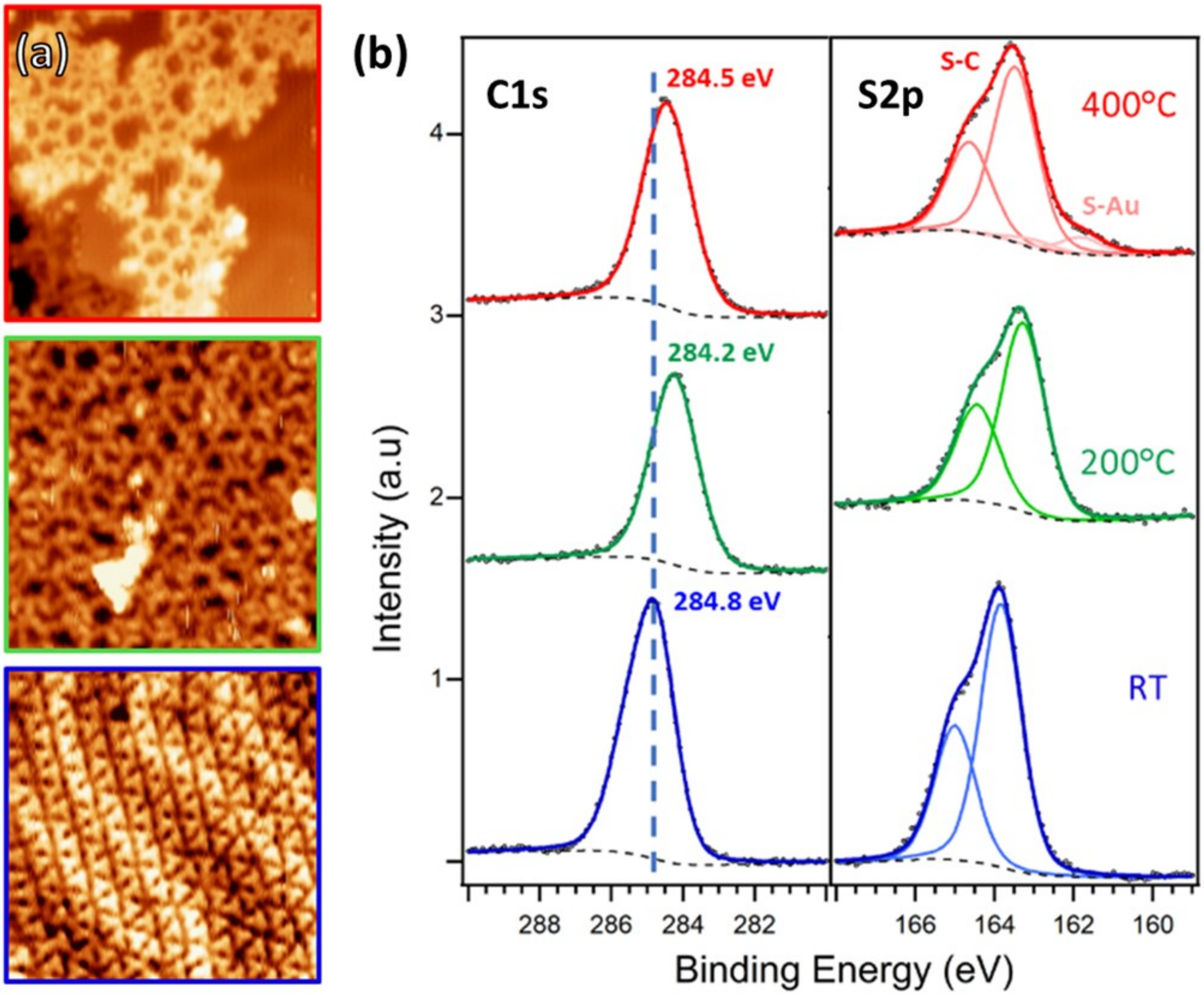

Figure S18. STM images (a) and XPS C 1s and S 2p spectra (b) of TBTTB deposited on Au(111) at RT (blue), and sequentially annealed to 200 °C (green) and 400 °C (red).

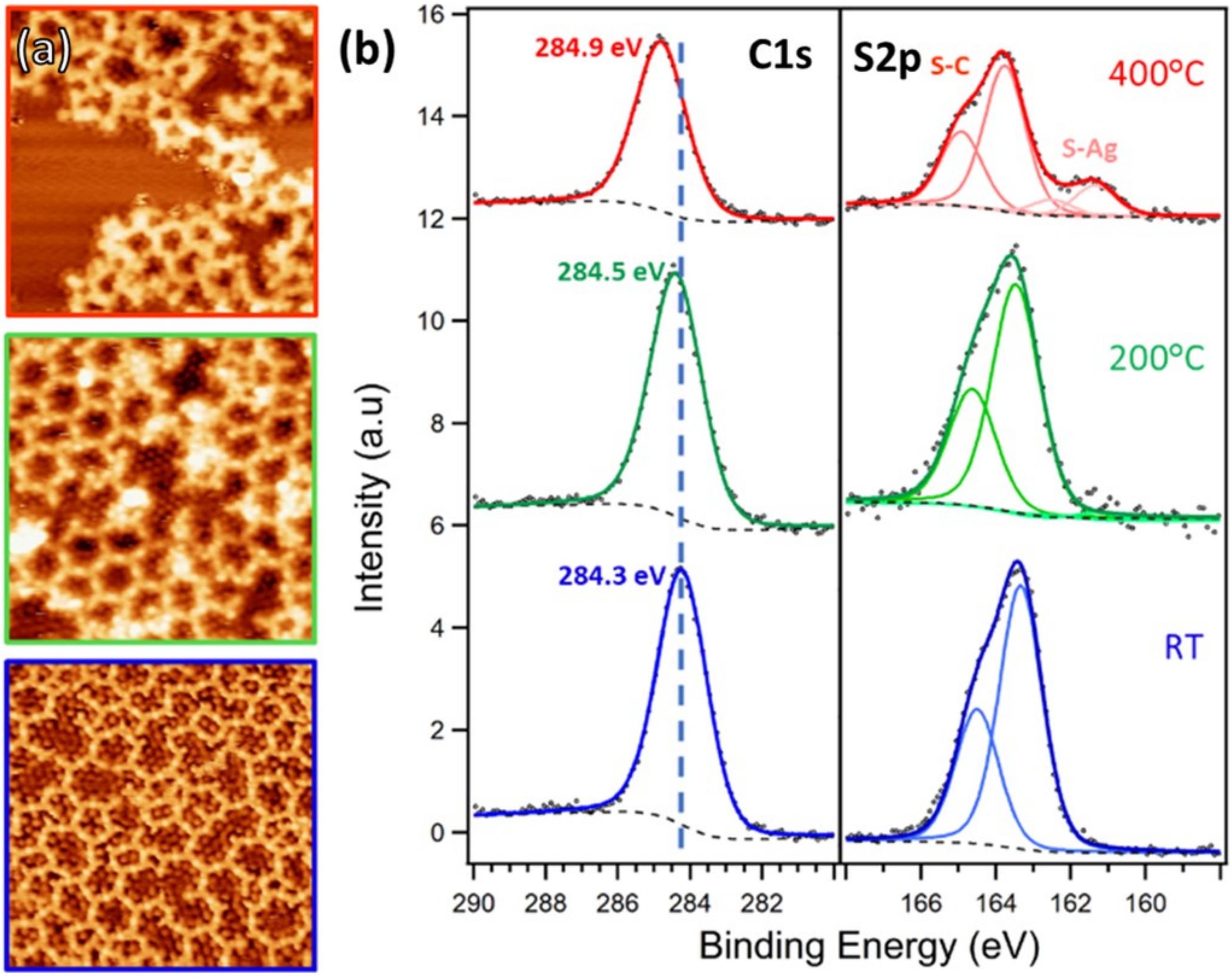

Figure S19. STM images (a) and XPS C 1s and S 2p spectra (b) of TBTTB deposited on Ag(111) at RT (blue), and sequentially annealed to 200 °C (green) and 400 °C (red).

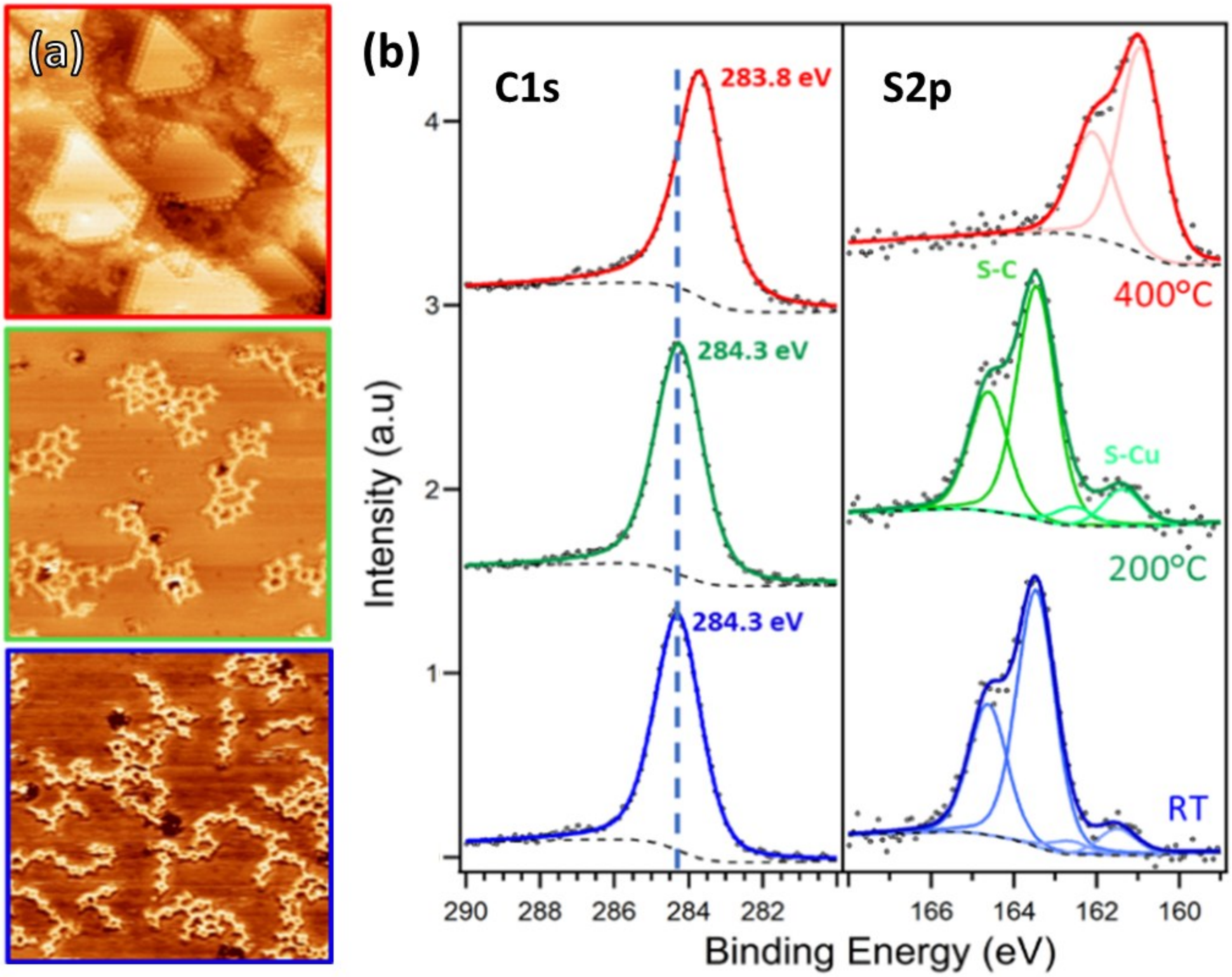

Figure S20. STM images (a) and XPS C 1s and S 2p spectra (b) of TBTTB deposited on Cu(111) at RT (blue), and sequentially annealed to 200 °C (green) and 400 °C (red).

8. Bibliography

1. Gutzler, R.; Fu, C.; Dadvand, A.; Hua, Y.; MacLeod, J. M.; Rosei, F.; Perepichka, D. F., Halogen bonds in 2D supramolecular self-assembly of organic semiconductors. *Nanoscale* **2012,** *4* (19), 5965-5971.
2. Pham, T. A.; Song, F.; Nguyen, M.-T.; Li, Z.; Studener, F.; Stöhr, M., Comparing Ullmann Coupling on Noble Metal Surfaces: On-Surface Polymerization of 1,3,6,8-Tetrabromopyrene on Cu(111) and Au(111). *Chem. - Eur. J* **2016,** *22* (17), 5937-5944.
3. Saywell, A.; Greń, W.; Franc, G.; Gourdon, A.; Bouju, X.; Grill, L., Manipulating the Conformation of Single Organometallic Chains on Au(111). *J. Phys. Chem. C* **2014,** *118* (3), 1719-1728.
4. Iski, E. V.; Jewell, A. D.; Tierney, H. L.; Kyriakou, G.; Sykes, E. C. H., Organic thin film induced substrate restructuring: An STM study of the interaction of naphtho[2,3-a]pyrene Au(111) herringbone reconstruction. *J. Vac. Sci. Technol.* **2011,** *29* (4), 040601.
5. Maksymovych, P.; Sorescu, D. C.; Yates, J. T., Gold-Adatom-Mediated Bonding in Self-Assembled Short-Chain Alkanethiolate Species on the Au(111) Surface. *Phys. Rev. Lett.* **2006,** *97* (14), 146103.
6. Frisch, M.; Trucks, G.; Schlegel, H. B.; Scuseria, G.; Robb, M.; Cheeseman, J.; Scalmani, G.; Barone, V.; Mennucci, B.; Petersson, G., Gaussian 09, revision a. 02, gaussian. *Inc., Wallingford, CT* **2009,** *200.*
7. Ourdjini, O.; Pawlak, R.; Abel, M.; Clair, S.; Chen, L.; Bergeon, N.; Sassi, M.; Oison, V.; Debierre, J.-M.; Coratger, R.; Porte, L., Substrate-mediated ordering and defect analysis of a surface covalent organic framework. *Phys. Rev. B* **2011,** *84* (12), 125421.
8. Billia, B.; Jamgotchian, H.; Nguyen Thi, H., Statistical analysis of the disorder of two-dimensional cellular arrays in directional solidification. *Metall. Mater. Trans. A* **1991,** *22* (12), 3041-3050.
9. Wallet, F.; Dussert, C., Comparison of spatial point patterns and processes characterization methods. *Europhys. Lett.* **1998,** *42* (5), 493.
